# Tumor Infiltrating Lymphocytes Target HLA-I Phosphopeptides Derived From Cancer Signaling in Colorectal Cancer

**DOI:** 10.3389/fimmu.2021.723566

**Published:** 2021-08-24

**Authors:** Sarah A. Penny, Jennifer G. Abelin, Stacy A. Malaker, Paisley T. Myers, Abu Z. Saeed, Lora G. Steadman, Dina L. Bai, Stephen T. Ward, Jeffrey Shabanowitz, Donald F. Hunt, Mark Cobbold

**Affiliations:** ^1^School of Immunity and Infection, University of Birmingham, Birmingham, United Kingdom; ^2^Department of Chemistry, University of Virginia, Charlottesville, VA, United States; ^3^Department of Colorectal Surgery, Queen Elizabeth Hospital, Birmingham, United Kingdom; ^4^Department of Pathology, University of Virginia, Charlottesville, VA, United States; ^5^Center for Cancer Immunology, Massachusetts General Hospital, Charlestown, MA, United States

**Keywords:** Immunopeptidomics, HLA-I phosphopeptides, tumor antigens, TIL (tumor infiltrating lymphocytes), CRC (colorectal cancer), signaling pathway

## Abstract

There is a pressing need for novel immunotherapeutic targets in colorectal cancer (CRC). Cytotoxic T cell infiltration is well established as a key prognostic indicator in CRC, and it is known that these tumor infiltrating lymphocytes (TILs) target and kill tumor cells. However, the specific antigens that drive these CD8+ T cell responses have not been well characterized. Recently, phosphopeptides have emerged as strong candidates for tumor-specific antigens, as dysregulated signaling in cancer leads to increased and aberrant protein phosphorylation. Here, we identify 120 HLA-I phosphopeptides from primary CRC tumors, CRC liver metastases and CRC cell lines using mass spectrometry and assess the tumor-resident immunity against these posttranslationally modified tumor antigens. Several CRC tumor-specific phosphopeptides were presented by multiple patients’ tumors in our cohort (21% to 40%), and many have previously been identified on other malignancies (58% of HLA-A*02 CRC phosphopeptides). These shared antigens derived from mitogenic signaling pathways, including p53, Wnt and MAPK, and are therefore markers of malignancy. The identification of public tumor antigens will allow for the development of broadly applicable targeted therapeutics. Through analysis of TIL cytokine responses to these phosphopeptides, we have established that they are already playing a key role in tumor-resident immunity. Multifunctional CD8+ TILs from primary and metastatic tumors recognized the HLA-I phosphopeptides presented by their originating tumor. Furthermore, TILs taken from other CRC patients’ tumors targeted two of these phosphopeptides. In another cohort of CRC patients, the same HLA-I phosphopeptides induced higher peripheral T cell responses than they did in healthy donors, suggesting that these immune responses are specifically activated in CRC patients. Collectively, these results establish HLA-I phosphopeptides as targets of the tumor-resident immunity in CRC, and highlight their potential as candidates for future immunotherapeutic strategies.

## Introduction

Tumor infiltration by effector T cells has been confirmed by several large studies to be significantly associated with good prognosis in CRC, even in metastatic disease (1–5). Cytotoxic T cells recognize antigenic peptides presented by HLA-I complexes on the surface of cancer cells. These T cells release cytotoxic factors, which kill the transformed cells and thus can control tumor growth. However, the targets of CD8+ T cells are not yet well defined in CRC (6).

The use of immunotherapies, such as immune checkpoint blockade (ICB), is revolutionizing cancer treatment, but their use has been limited in the majority (86%) of CRC, which are microsatellite stable (MSS) (7). The remaining 14% of tumors that may respond to ICB therapies, have a defect in DNA mismatch repair, leading to microsatellite instability (MSI) and thus a high tumor mutational burden (TMB) (8, 9). Irrespective of therapy, MSI is predictive of favorable outcomes in CRC, associated with the extensive cytotoxic T cell infiltration into these tumors (9, 10); however, once the tumor has evaded T cell recognition and recurrence occurs, prognosis is poor (7). Preclinical studies have demonstrated that PD-1 blockade is only effective in the presence of fully primed and committed antigen-specific T cells (11). Therefore, it has been proposed that the T cells infiltrating MSI CRC are targeting the mutational neoantigens that arise from the high TMB in these tumors (12, 13). TMB does show a strong correlation with ICB-response (14, 15), yet it has proven controversial and challenging to implement as a response biomarker in clinical practice (16). Moreover, cytotoxic T cell infiltration has also been shown to be a significant predictor of prognosis in MSS CRC, even though these usually have a low to moderate TMB (9). Whilst some TILs may target mutational neoantigens in MSS CRC, it seems unlikely that T cell targeting of these limited antigens could fully explain the control of tumor growth in CRC; consequently, other classes of tumor antigens are almost certainly implicated (6).

Mutations in the Wnt, TGF-β and RAS signaling pathways are ubiquitous in CRC, often affecting critical kinases and phosphatases (17). Deregulation of these kinases can lead to the activation of several signaling cascades and increase the extent of protein phosphorylation within tumor cells (18). Therefore, we hypothesized that the infiltrating cytotoxic T cells in CRC may be targeting, not only mutated peptides, but also the phosphorylated peptides resulting from this dysregulated signaling. Phosphorylated peptides have already been defined as strong candidates for tumor-specific antigens in melanoma, renal cell carcinoma and hematological malignancies (19–21). Phosphorylation is preserved on peptides through antigen processing and presentation pathways (22), and can produce T cell epitopes that differ structurally from their unphosphorylated counterparts (23, 24). In some instances, this has been shown to be due to phosphorylation-induced conformational alterations in the peptide, facilitated by novel contacts with the HLA complex (23, 25). Yet, even when these conformational alterations are minimal, T cell receptor (TCR) recognition can be specific to the phosphorylated peptide form (23). These data suggest that phosphopeptide specific T cells may not be deleted by central tolerance in the same way as those targeting other non-mutational tumor-associated antigens (26). Phosphopeptide antigens also provide an advantage as potential immunotherapeutic targets because they are often shared across patients (19). We hypothesized that CRC-specific phosphopeptides may represent a subset of posttranslationally modified (PTM) tumor antigens targeted by the tumor-resident CD8 T cell response in CRC.

Previous efforts in defining the CRC immunopeptidome have focused on mutational neoantigens and cancer germline antigens (6). To identify putative PTM tumor associated antigens, we used CRC cell lines, primary CRC tumors and CRC liver metastases. From these we purified HLA-I peptides, enriched for phosphopeptides and then sequenced these using mass spectrometry (MS). We evaluated the responses of TILs taken from the same tumors that were used for phosphopeptide identification, and then broadened this to determine if the immunogenic phosphopeptides may represent public T cell targets in CRC. In addition, peripheral T cell responses targeting CRC phosphopeptides were compared between patients and healthy donors. Finally, the functional capacity of the TILs targeting phosphopeptides was assessed in killing assays. We hope that identifying these PTM tumor antigens, and understanding their role in tumor immunity, may support the development of future targeted tumor immunotherapies.

## Materials And Methods

### Patient and Healthy Donor Samples and Cell Lines

Tumor and blood samples were obtained fresh from University Hospital Birmingham. Samples were received from the Human Biomaterials Resource Centre (HBRC) at the University of Birmingham (Ref 09/H1010/75) and *via* studies approved by research ethics committees local to the University of Birmingham (Ref: 06/Q2702/61 and 09/H1203/49). Informed written consent was obtained in accordance with the Declaration of Helsinki, in all cases. Fresh blood samples were collected from patients and healthy donors. 20-30 mL of blood was collected in vacutainers containing lithium heparin (BD, UK). CRC cell lines were grown up from laboratory stocks, originating from the European Collection of Cell Cultures and the American Type Culture Collection. All cells were stored in fetal bovine serum (FBS) with 10% DMSO at -80°C. The cells were managed in sterile conditions in a laminar airflow hood, at all times, and incubated at 37°C with 5% CO_2_. Cell lines were grown in D-MEM (Dulbecco’s Modified Eagle Medium (1X) liquid (High Glucose), 10% FBS, 1% penicillin/streptomycin) and the cells were grown in 25 cm^2^, 75 cm^2^, 175 cm^2^ and 1720 cm^2^ vented tissue culture flasks, with 20 mM HEPES buffer added. The cells were observed using a phase contrast microscope. Cell counts and viability were determined by the exclusion of Trypan blue dye by light microscopy. Mycoplasma infection was excluded in all cell lines, using the Lonza MycoAlert Mycoplasma Kit (Lonza, ME, USA).

### TIL Isolation and Expansion

In a laminar flow hood, the tumor was sliced into small (2 mm x 2 mm) chunks and each one placed into 2 mL T cell medium, containing extra antibiotics to eliminate gut bacteria and yeast (AIM-V/10% Human serum/25 mM HEPES/6000 IU/mL IL-2/50 μg/mL neomycin/2 μg/mL micofungin/55 µM β-mercaptoethanol/15 μg/mL metronidazole/20 μg/mL vancomycin). The TILs were maintained at 1x10^6^ cells/mL in T cell medium in a 24-well plate for 14-21 days. The cells were subsequently FACS analyzed to determine the proportion of CD8+ TILs and aliquots frozen.

TILs were rapidly expanded using a standard rapid expansion protocol (REP), as described by Dudley and colleagues (27). Briefly, 1x10^6^ thawed TILs were added to REP medium (2x10^8^ irradiated PBMCs/30 ng/mL OKT3/6000 IU/mL IL-2/50 mL RPMI 10% HS/50 mL AIM-V/50 μg/mL neomycin/2 μg/mL micofungin/55 µM β-mercaptoethanol/25 mM HEPES). Half of the medium was exchanged on day 5. On day 7 the TILs were counted and their density reduced to 0.5 million/mL. Cells were maintained at 0.5 million/mL until day 14 when aliquots of up to 30 million TILs were frozen.

### Isolation of HLA-Associated Peptides

HLA class I molecules were immunoaffinity purified from samples, and their associated peptides were extracted as described previously (22, 28). Briefly, 1x10^9^ cells, or 1 g of tissue were lysed in 10 mL of 20 mM Tris-HCl pH 8.0/150 mM NaCl with 1% 3-[(3-cholamidopropyl) dimethylammonio]-1-propane sulfonate (CHAPS)/1 mM PMSF/5 μg/mL aprotinin/10 μg/mL leupeptin/10 μg/mL pepstatin A/and phosphatase inhibitor cocktails II and III (Sigma-Aldrich, UK) at 4°C. Tissues were homogenized, on ice. The lysate was subject to ultra-centrifugation at 100,000 *x g*, for 1 hour, at 4°C (Optima LE-8K (Ti70 rotor), Beckman-Coulter, UK). Supernatants were incubated overnight with W6/32 antibody-bound NHS sepharose beads, specific for HLA class I molecules (Amersham Pharmacia Biotech). The beads then underwent a series of washes in lysis buffer, TBS (20mM Tris-HCl/150 mM NaCl pH 8), TBS2 (20 mM Tris-HCl/1 M NaCl pH 8), and 20 mM Tris-HCl pH 8. Peptides were eluted from the MHC class I molecules with 10% acetic acid and isolated by ultrafiltration (Ultrafree-MC, Millipore).

### HLA-I Phosphopeptide Enrichment by IMAC

As previously described (29), samples were initially passed through C18 microcapillary cleanup-columns to desalt, internal standards were added and the samples dehydrated. Prior to phosphopeptide enrichment using Fe^+3^-immobilized metal affinity chromatography (IMAC), peptides were esterified to neutralize acidic groups on the peptides and prevent non-specific binding (29). IMAC columns, created in-house, as described previously (29), were used to enrich for phosphopeptides. These were eluted directly onto a C18 precolumn, and transferred to an analytical column containing irregular C18 packing material equipped with an electrospray emitter tip. Two further internal standard peptides, were loaded prior to analysis.

### HPLC-ESI-MS/MS Analysis of IMAC Enriched Phosphopeptides

Enriched phosphopeptides were gradient eluted through an electrospray tip directly into a Thermo Scientific LTQ-FT-ion cyclotron resonance mass spectrometer equipped with an Agilent 1100 series binary HPLC. Mass spectra were acquired in the high-resolution Fourier transform mass analyzer, and the tandem MS spectra were acquired in the linear ion trap of the LTQ-FT-ion cyclotron resonance instrument using collision-activated dissociation (CAD) and electron transfer dissociation (ETD). Detailed methods have been previously described (29).

Data analysis was performed by using the Xcalibur software (Thermo Electron Corporation). The data files were searched against the RefSeq database (downloaded June 2009) using OMSSA (version 2.1.1) (30). An in house software program called “Neutral Loss Finder” was also used to identify phosphopeptides from their neutral loss of phosphoric acid (98 Da) in MS2 CAD spectra. OMSSA and neutral loss search results were used to guide the analysis, but all peptide sequences were determined by accurate mass measurement and manual interpretation of the MS2 spectra using theoretical peptide fragment ion masses (**Supplementary Figure 1**).

### HLA Typing and Epitope Prediction

HLA sequences were obtained to 2 digits from the NHS clinical facility. The MHC-I binding predictions were made on 7/22/2017 using the IEDB analysis resource Consensus tool and the 6 predetermined HLA alleles for each patient (31).

### Intracellular Cytokine Staining

CD8+ T cells were extracted from frozen stocks using magnetic cell separation (Miltenyi Biotec, Germany). TIL responses were expanded over 6 days. 100,000 irradiated CD8- TILs/well in TIL medium (50% AIM-V, 40% RPMI, 10% Human serum, 100 IU/mL penicillin, 100 μg/mL streptomycin, 100 mg/mL Neomycin, 2 mg/mL Micafungin, 7.5 μg/mL metronidazole, 2000 IU/mL IL-2) were pulsed with 10 μg/mL peptide overnight. These were washed and added to 200,000 CD8+ TILs/well of a 48-well plate in TIL medium. The positive control was stimulated with 1 μg/mL PHA. On day 6 the TILs were all washed twice in AIM-V and resuspended in TIL medium containing 5 μg/mL of Brefeldin A and monensin. Each well was restimulated overnight with CD8- TILs that had been peptide pulsed for 2 hours with 10 μg/mL of the relevant peptide. They were then harvested, washed with PBS and stained with a fixable viability dye (APC-Cy7) (eBiosciences) and the surface antibodies, anti-CD3 (APC) and anti-CD8 (PerCP) (Biolegend, Cambridge, UK). The cells were then washed in MACS buffer, fixed, using 2% paraformaldehyde, washed, permeablized using 0.5% saponin, and stained with anti-IFNγ (PE), anti-IL-2 (Pacific blue) and anti-TNFα (PE-Cy5.5) (BioLegend, Cambridge, UK) for 30 minutes at room temperature. Cells were washed again with MACS buffer and lightly fixed until they could be analyzed on the BD LSR-Fortessa flow cytometer (BD, Oxford, UK).

### Immunohistochemistry

Sections of formalin fixed paraffin embedded tumor were baked at 60°, deparaffinized in xylene and rehydrated in descending gradient alcohols. The Leica Bond-max was used for staining, alongside Leica antibodies (Leica, UK). Endogenous peroxidase activity was blocked by incubation with 3% hydrogen peroxide for 5 min. Mouse monoclonal anti-human antibodies (Leica, UK) were applied as per manufacturer’s instructions. Sections were then incubated with HRP polymer for 8 min, washed and then developed for 10 min using diaminobenzidine (DAB) solution. The sections were counterstained in hematoxylin, dehydrated in alcohol and xylene and mounted using an automated coverslipper (Leica, Germany).

### Enzyme-Linked Immunospot Assay

A cultured ELISpot was used, as previously described (19). PBMCs were cultured in AIM-V medium (Invitrogen) for 6 days with 2 ug/mL peptide at 5x10^5^/mL. The ELISpot PRO for human IFNγ kit (Mabtech, Sweden) was used for these experiments. On day 6 cells were washed and transferred in AIM-V to the pre-washed polyvinylidene difluoriden (PVDF)-backed microplates coated with a monoclonal antibody specific for human IFNγ. The cells were restimulated with the relevant peptide at 2 µg/mL, and the CD3 monoclonal antibody (100 ng/mL) added to the positive control. The microplate was incubated for overnight at 37°C. The cells were then removed and the plate treated as per manufacturer’s instructions. Once dry, the spots representing individual IFNγ secreting cells were counted using an ELISpot reader (AID ELISpot Reader HR XL, Advanced Imaging Devices GmbH, Germany) and images captured using the AID ELISpot Software 4.0 (AID GmbH, Germany).

### Generation of Phosphopeptide-Specific T Cell Lines

CD8+ TILs were plated at 1x10^6^/mL in TIL medium and stimulated with 10 μg/mL of the relevant peptide. On days 7 and 10 TILs were adjusted to 5x10^5^/mL and half of the medium exchanged. On day 14 phosphopeptide-specific TILs were selected, using CD137 MACS (Miltenyi biotech). Half of the cells were peptide pulsed for 2 hours, washed and added to the other half overnight. These were then rapidly expanded, and used in a killing assay on day 24.

### Killing Assay

A Europium release assay was used, which produces results that are similar to the classical chromium release assay (32). Target cells were washed in the relevant medium and resuspended to 1 million cells/mL. 2.5 μL/mL of the BATDA fluorescence enhancing ligand was added and the cells incubated for 30 minutes at 37°C, 5% CO2 in a humidified environment. The cells were washed five times in excess medium. 10,000 target cells were added to each well of a V-bottomed 96-well plate. T cells at varying effector to target (E:T) ratios were added to the test wells. Lysis buffer was added to the wells for maximal release. All well volumes were made up to 200 μL. The plate was incubated for 2-5 hours in a humidified 5% CO2 atmosphere at 37°C. 20 μL of each supernatant was transferred to a flat-bottomed, white, 96-well plate and 200 μL of Europium solution was added. This was incubated for 15 minutes, shaking, at room temperature. The fluorescence was measured in a time-resolved fluorometer (Tecan Infinite 200 PRO; Tecan, Switzerland).

### Statistical Analysis

Statistical analysis was performed using Graphpad Prism 5.0. EulerAPE was used to produce the Euler diagrams (33). Weblogo was used to generate the sequence logos (34).

## Results

### Tumor-Specific HLA-I Phosphopeptides Are Presented by CRC Cell Lines and Tumors

**Figure 1:**
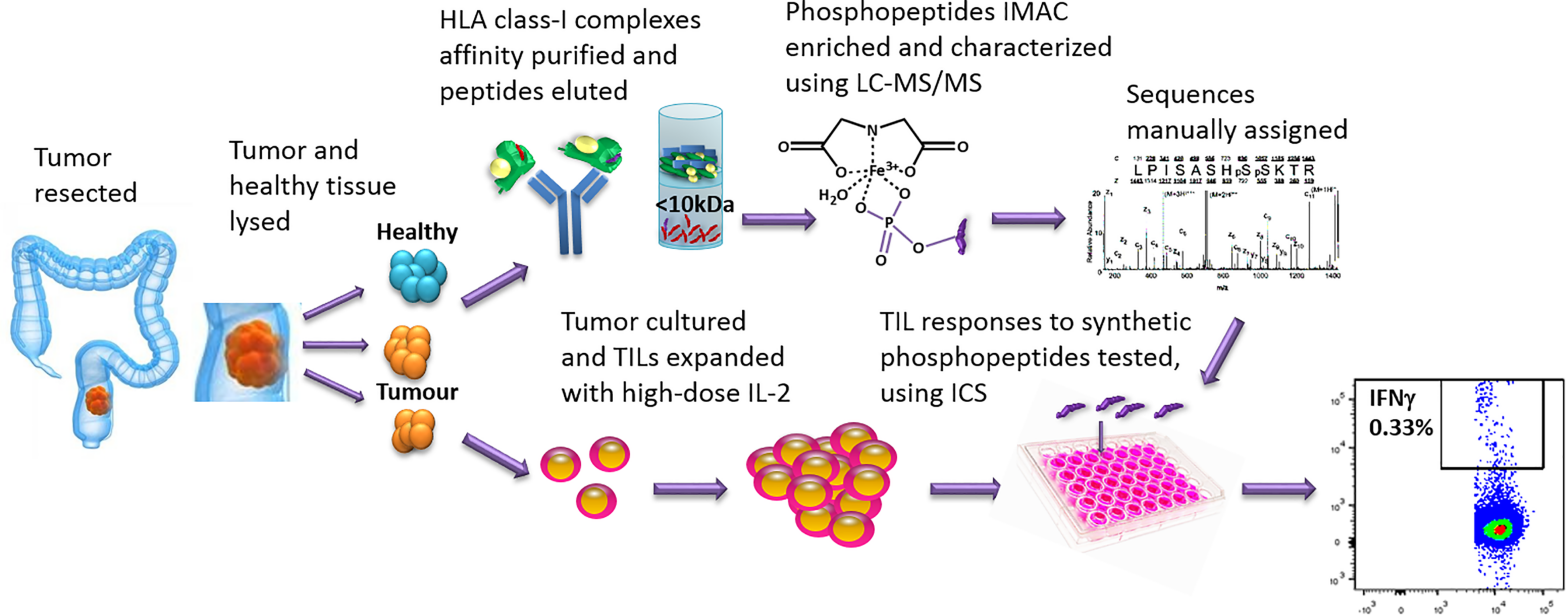
The work-flow used to identify phosphopeptides from CRC tumors and to test TIL responses to the phosphopeptides identified. Tumor and neighboring healthy tissue samples were taken at resection. Some tumor was cultured and the TILs expanded, using high-dose IL-2. The remaining sample was homogenized in sample buffer and HLA-I complexes affinity purified with immobilized W6/32 antibody. The peptides were acid eluted, the phosphopeptides IMAC enriched and then characterized using LC-MS/MS. Synthetic phosphopeptides were used to test TIL responses to the phosphopeptides identified on the tumors using an intracellular cytokine staining assay.

**Figure 2:**
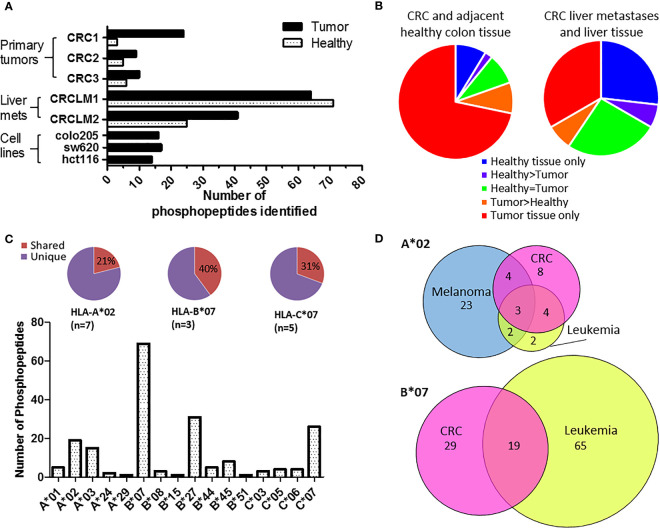
Phosphopeptides identified from colorectal cancer samples – putative cancer antigens. **(A) **Comparison of phosphopeptides identified on tumors and healthy tissue from CRC patients with primary (n=3) and secondary (n=2) tumors and CRC cell lines (n=3). **(B)** Proportion of the phosphopeptides identified on primary and secondary tumors that were healthy specific (not detected on tumor) (blue), healthy associated (more detected on healthy than tumor tissue) (purple), equal (where equal was within one order of magnitude) (green), tumor associated (more detected on tumor than healthy tissue) (orange) and tumor specific (not detected on healthy tissue) (red). **(C)** The number of phosphopeptides identified predicted to bind to different HLA-I. Pie charts show the proportion of phosphopeptides shared (red) across multiple patients’ samples for the most common HLA-alleles. **(D)** Overlap of HLA-A*02 and HLA-B*07 phosphopeptides identified on CRC (pink) with those found on other types of malignancy; melanoma (blue) and leukemia (yellow).

To identify tumor-specific phosphopeptides, we compared HLA-I phosphopeptides found on healthy colon tissue to those on tumor tissue. HLA-peptide complexes were affinity purified, the HLA-bound peptides eluted, and immobilized metal affinity chromatography (IMAC) was used to enrich for phosphopeptides, which were identified using liquid chromatography with tandem mass spectrometry (LC-MS/MS) (**Figure 1** and **Supplementary Figure 1**). Three primary tumors, two liver metastases and three CRC cell lines were used to identify a total of 198 phosphopeptides, of which 120 were tumor-associated in our cohort: 74 tumor-specific (identified only on tumor), 12 tumor-associated (at least 10-fold higher amounts identified on tumor than on healthy tissue), and 34 CRC cell line-associated phosphopeptides (**Supplementary Tables 1**–**5**). More phosphopeptides were identified on primary tumors than neighboring healthy colon tissue – 3.1 fold more, with 73% of phosphopeptides being assigned as tumor-specific (**Figures 2A, B**). Liver metastases presented even more tumor-specific phosphopeptides − 1.5 fold the number identified on the primary CRC tumors. Many more phosphopeptides were also seen in neighboring healthy liver tissue than healthy colon tissue, so the number of phosphopeptides identified on healthy liver tissue and CRC liver metastases were comparable (**Figure 1B**).

The phosphopeptides identified were predominately nine (63%) or ten (23%) amino acids long and contained phosphoserine (92%) at position four (71%) (**Supplementary Figure 2**). These phosphopeptides were predicted to be presented by 16 different HLA alleles, but nonetheless 23% were shared across multiple patients’ samples in our cohort (**Figure 2C** and **Supplementary Figure 3**; **Supplementary Tables 3–5**). This proportion increased to 40% when looking at HLA-B*07, a commonly expressed HLA allele (n=3) that presents many phosphopeptides (**Figure 2C** and **Supplementary Figure 4**). The list of CRC-associated phosphopeptides was compared with published lists of leukemia and melanoma phosphopeptides (19, 20). Many of the CRC phosphopeptides have been identified on other malignancies - 11/19 (58%) of HLA-A*02, and 19/48 (40%) of HLA-B*07 phosphopeptides, with a subset being shared across all malignancies tested (**Figure 2D** and **Supplementary Table 6**). These shared epitopes may be representative of mitogenic signaling pathways shared across cancers. Therefore, we mapped the proteins to known CRC oncogenic pathways (35), and demonstrated that many of the tumor-specific HLA-I phosphopeptides derive from proteins involved in key signaling pathways, such as Wnt, MAP kinase, TGFβ and p53 (**Supplementary Figure 5**). Although we have denoted many of the phosphopeptides as tumor-specific, because we could not identify them on healthy tissue, there may be small amounts present on healthy cells that are below the limits of detection in mass spectrometry, or on other tissues that were not sampled. Regardless, the detection of PTM tumor antigens, which are associated with key signaling pathways, on CRC tumors hinted that they could be targeted by TILs.

### Patient TILs Target Tumor-Specific HLA-I Phosphopeptides

**Figure 3:**
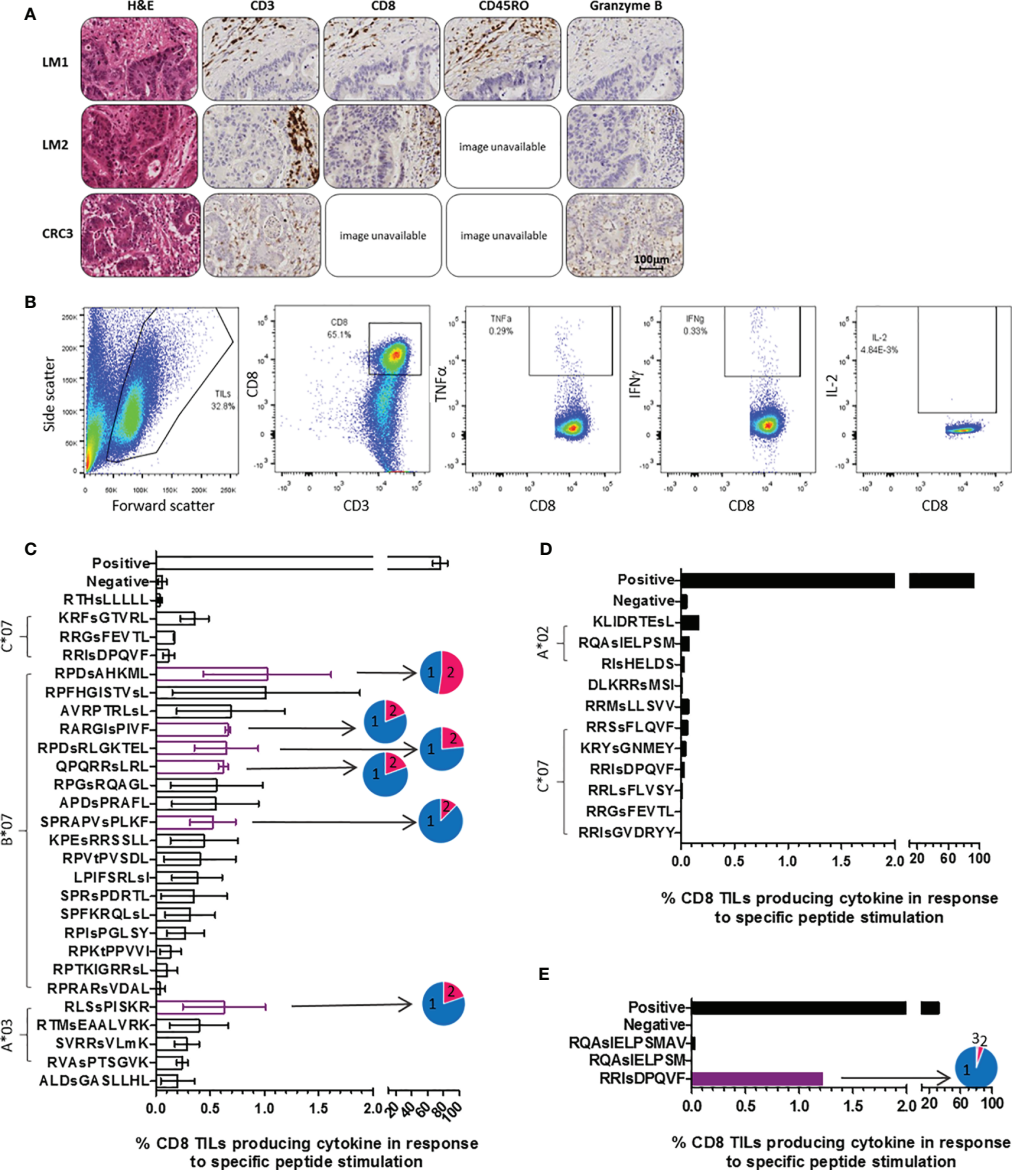
TILs target phosphopeptides found on CRC tumors. **(A) **Immunohistochemistry (IHC) of the tumors that TILs were taken from, stained for CD3, CD8, CD45RO and Granzyme B (DAB staining in brown). Some slide images unavailable due to loss of section. **(B)** ICS gating strategy utilized in assessment of TIL phosphopeptide responses. **(C) **CRCLM1 TIL cytokine responses to phosphopeptides identified on CRCLM1 (results represent the assay repeated on 2 separate occasions, with differentially expanded TILs), pie charts show the proportion of T cells producing 1 (blue), 2 (pink) and 3 (purple) cytokines. Consistently strong responses are highlighted in purple. **(D)** CRCLM2 TIL cytokine responses to phosphopeptides identified on CRCLM2. **(E)** CRC3 TIL cytokine responses to phosphopeptides identified on CRC3, pie chart shows the proportion of T cells producing 1 (blue), 2 (pink) and 3 (purple) cytokines.

Immunohistochemical analysis of patient tumors revealed T cell infiltration of the stroma in all cases, with functional cytotoxic T cell infiltration of the tumor apparent in the primary CRC tumor (**Figure 3A**). To assess whether patients harbored tumor-resident T cells capable of targeting the HLA-I phosphopeptides identified on autologous CRC tumor, TILs were extracted, expanded using cytokines, and frozen (27). Once the phosphopeptide analyses were complete, we selected the tumor-associated and tumor-specific phosphopeptides binding common HLA alleles for further study. After a 6-day culture with peptide, we quantitated immunity within the TILs against phosphopeptides, or control viral peptides (19), using intracellular cytokine staining (ICS) (**Figure 3B** and **Supplementary Figure 6**). We observed that multifunctional, memory TILs target many of the phosphopeptides identified in a CRC liver metastasis sample (CRCLM1) and a single phosphopeptide in primary CRC sample - CRC3 (RRIsDPQVF, which was predicted to bind HLA-C*06 in this patient), producing TNFα, IFNγ, and IL-2, but no TIL responses targeting phosphopeptides were detected in CRCLM2 (**Figures 3C–E** and **Supplementary Figures 6–8**). Considerable variability was observed in TIL responses, but despite this variation, clear TIL responses to a subset of the LC-MS/MS detected HLA phosphopeptides were seen. Surprisingly, phosphopeptides that were also present on adjacent healthy tissue could be targeted by TILs. Namely, those targeting RRIsDPQVF in CRC3, which was found at 18 times greater concentration on tumor than healthy colon tissue (**Supplementary Table 5** and **Figure 3E**), and two phosphopeptides identified on CRCLM1, though these were identified only at very low concentrations (<1fmol/g) on neighboring healthy liver tissue (**Supplementary Figure 9**). In this small cohort, all TIL responses to the HLA-I phosphopeptides were unique; therefore, we expanded our cohort of patients to investigate whether TIL responses to shared HLA-I phosphopeptides could be found more broadly in CRC patients.

### T Cells Targeting CRC HLA-I Phosphopeptides Are Present in CRC Patient Tumors and Peripheral Blood

**Figure 4:**
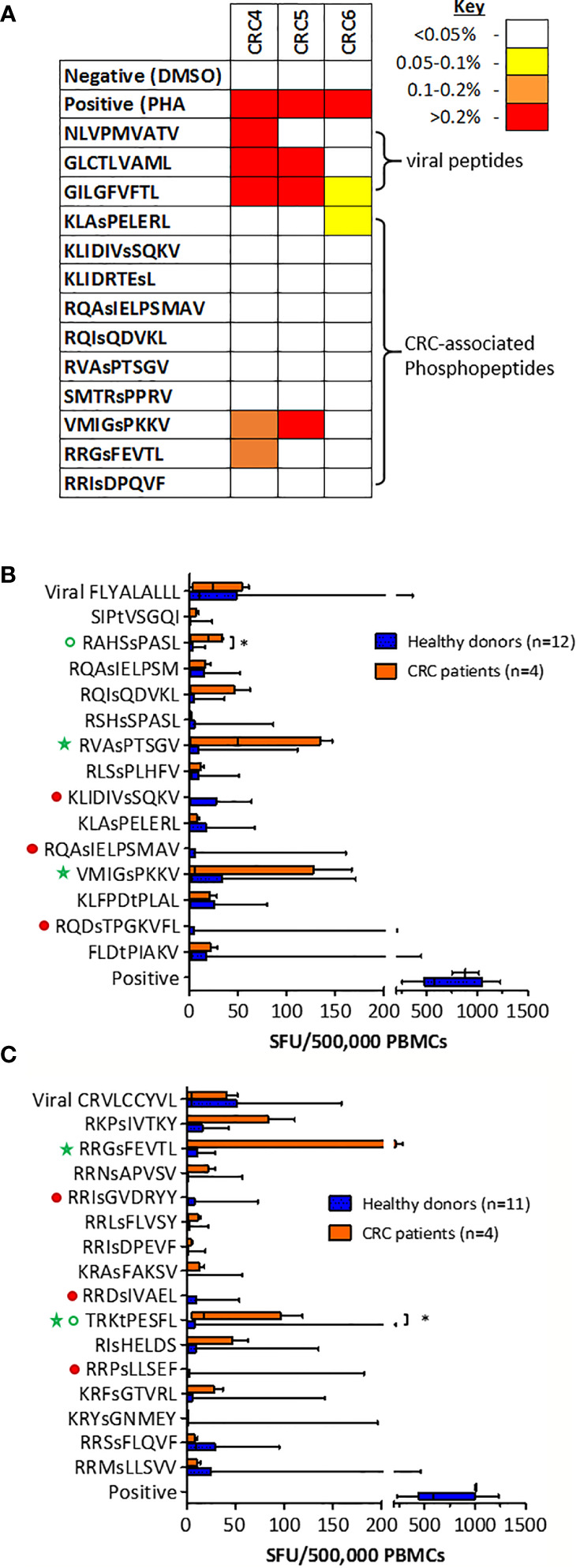
T cells targeting CRC phosphopeptides are found in a number of patients. **(A)** Summary of CRC TIL cytokine responses to common phosphopeptides (n=3). Box and whisker plots comparing HD and CRC patient PBMC IFNg production targeting **(B)** HLA-A*02-associated phosphopeptides and **(C)** HLA-C*07-associated phosphopeptides; median, interquartile range (box) and minima to maxima (whiskers) are shown. Responses that were absent (red circles) in CRC patients are highlighted. Green circles highlight the responses that were significantly higher in CRC patients than HD (Mann-Whitney U, * denotes p<0.05). Green stars highlight the phosphopeptides to which at least one patient had a high PBMC response.

We have demonstrated that the same tumor-specific HLA-I phosphopeptides are presented on CRC tumors from different patients, and also on other malignancies. To evaluate if any CRC phosphopeptides are commonly targeted by CRC patient TILs, we selected a subset of phosphopeptides that were predicted to bind to commonly expressed HLA-I alleles - HLA-A*02, HLA-C*06 and HLA-C*07 - and have also been identified in other malignancies (19, Penny et al., unpublished). TIL responses were assessed, using ICS, in a small cohort of patients. In all of the patients tested, we found TILs targeting the CRC-associated HLA-I phosphopeptides, with moderate to strong responses against two peptides (**Figure 4A**). These TIL responses were detected against an HLA-A*02-associated phosphopeptide from tensin 3 (VMIGsPKKV) and an HLA-C*07-associated phosphopeptide from selenoprotein H (RRGsFEVTL), with responses comparable in magnitude to those targeting control viral antigens. Thus, we identified shared tumor-specific phosphopeptide antigens on CRC and demonstrate that these same phosphopeptides are targeted by TILs in tumors whose HLA peptidomes have not been characterized by LC-MS/MS.

To investigate whether the T cells that target HLA-I phosphopeptides could be found in the peripheral blood of CRC patients, we used cultured IFNγ ELISpot (**Supplementary Figure 10**), and compared responses between CRC patients and healthy donors. We detected T cells targeting HLA-I phosphopeptides in the peripheral blood of CRC patients (**Figures 4B, C** and **Supplementary Figure 11**). Interestingly, responses targeting some of the CRC-associated HLA-I phosphopeptides were absent in CRC patients, but present in healthy donors, as has been seen in other malignancies (19, Buettner et al., unpublished) (**Figures 4B, C**, denoted by filled red circles). However, there were also HLA-I phosphopeptides that elicited higher responses in CRC patients than their healthy counterparts (**Figures 4B, C**, open green circles), some significantly so - namely RAHSsPASL (p=0.032) a peptide from transcriptional coactivator YAP1, from the TGFβ signaling pathway (**Figure S4**) and TRKtPESFL (p=0.036) a peptide from Epsin-1. Of note, strong responses could also be seen in the circulating T cells of many CRC patients targeting HLA-A*02-associated peptides RVAsPTSGV and VMIGsPKKV (**Figure 4B**), and HLA-C*07-associated peptide RRGsFEVTL (**Figure 4C** and **Table 1**). Although interpretation of these results is limited by the small patient cohort, the peripheral T cell responses observed align with responses seen in other patients at the tumor site, and warrant future investigation in additional CRC patients, as there appears to be a clear role for phosphopeptide-specific T cells in CRC.

**Table 1:** Summary of responses to key CRC HLA-I phosphopeptides.

Phosphopeptide	RVAsPTSGV	VMIGsPKKV	RRGsFEVTL
Protein of Origin	IRS2	TNS3	SELH
HLA-binding	A*02	A*02	C*07
Samples	colo205 & hct116	sw620	CRC1, CRCLM1, CRCLM2
Other malignancies	Melanoma & Leukemia	Melanoma & Leukemia	
TIL responses	–	+++	++
HD PBMC responses	++	+++	+
CRC patient PBMC	+++	+++	+++

+ low, ++ moderate, +++ strong responses, - not detected.

### Phosphopeptide-Specific T Cells Can Kill, but May Become Dysfunctional

**Figure 5:**
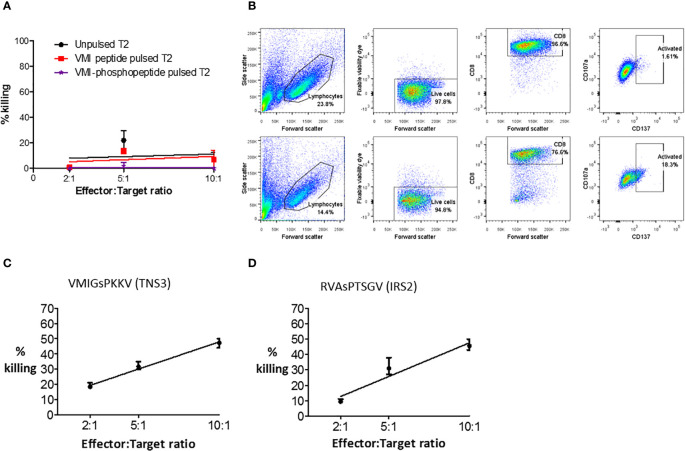
Phosphopeptide-specific T cells can kill, but expanded TILs do not. **(A)** A europium release assay, using a VMI-specific T cell line grown from patient (CRC5) TILs to target T2 cells pulsed with phosphopeptide VMIGsPKKV, or unphosphorylated VMIGSPKKV. **(B)** Flow cytometry to show the activation and degranulation of VMI-specific CRC5 TILs when stimulated with unphosphorylated VMIGSPKKV (top) and phosphorylated VMIGsPKKV (bottom). Healthy donor T cell lines targeting **(C)** VMIGsPKKV and **(D)** RVAsPTSGV were used in a europium-release killing assay with a CRC cell line natively expressing the phosphopeptides (SW620).

To test phosphopeptide-specific T cell functionality, T cell lines were established from primary CRC TILs and healthy donor peripheral blood mononuclear cells (PBMCs). Phosphopeptide-specific cells were enriched using magnetic cell sorting for activation marker CD137, after stimulation with phosphopeptide (**Supplementary Figure 12**). Surprisingly, when we evaluated cytotoxicity, the extensively expanded, phosphopeptide-specific TILs tested did not kill phosphopeptides pulsed target cells (**Figure 5A**). Nonetheless, some of the CD8^+^ TILs were activated in response to phosphopeptide, but not its unphosphorylated counterpart, with 16.7% upregulating the T cell activation marker CD137 (**Figure 5B**). Thus proving the TILs specifically target the phosphopeptide, and not the unmodified peptide. Unlike the TILs, T cell lines grown from healthy donor PBMCs targeting VMIGsPKKV and RVAsPTSGV were functional and did kill, in a dose-dependent manner (**Figures 5C, D** and **Supplementary Figure 13**). These observations suggest a loss of functional competence in HLA-I phosphopeptide specific T-cell responses - either at the tumor site, or due to their extensive *ex vivo* expansion – as phosphopeptide-specific T cells can clearly target and kill CRC cells.

## Discussion

Despite T cell infiltration of CRC being well-established as the most important predictor of prognosis in CRC, the targets of these T cells remain ill-defined (1, 3, 5, 6, 9). This has limited the development of antigen targeted immunotherapies in CRC, especially as the vast majority of tumors are MSS and have a low to moderate TMB (6). Recently, case reports have demonstrated promising proof of concept for adoptive T cell (ATC) therapy - targeting a HLA-C*08 restricted mutational neoantigen (KRAS G12D) in metastatic CRC - showing it to be both safe and effective (36). However, targeting mutational neoantigens is limited by the low frequencies of public neoantigens combined with the highly polymorphic nature of HLA alleles (37). Here, we have identified 74 tumor-specific HLA-I bound phosphopeptide antigens from primary and secondary CRC tumors, which may represent targetable PTM tumor antigens in CRC. We have shown that CRC TILs target these phosphopeptides, implicating them in tumor immunity and thus indicating that they may be favorable candidate immunotherapeutic targets. Importantly, although HLA-restricted, these PTM tumor antigens are public and consequently afford the opportunity to develop “off-the-shelf” cell therapy approaches. Future immunotherapeutic strategies could include: ATC therapy, using phosphopeptide-specific T cells expanded *ex vivo*, or engineered TCRs; chimeric antigen receptor (CAR) T cells; the use of bispecific TCRs targeting phosphopeptides; a phosphopeptide vaccine; or a combination therapy, including ICB (38–40).

Our data indicate that HLA-I phosphopeptides may be favorable candidate immunotherapeutic targets for several reasons. Firstly, we have shown a link between cancer signaling and HLA-I phosphopeptide presentation, in that some HLA-I phosphopeptides are tumor specific, and their numbers increase with tumor stage progression (**Figures 2A, B**). This mirrors an increase in aberrant signaling as cancers progress and metastasize (18). However, although the number and amount of phosphopeptides presented appeared to correlate with malignancy, these differences could also have been attributed to the specific binding preferences of the expressed HLA alleles. For example, we show that both HLA-B*07 and HLA-B*27 present more phosphopeptides than other alleles (**Figure 2C** and **Supplementary Figure 4A**), as previously reported (41). This overrepresentation could be ascribed to the similarity of the HLA- and the kinase- binding motifs (**Supplementary Figure 4B**). Nonetheless, of the two HLA-B*07 samples (CRC1 and CRCLM1, from different patients), the liver metastasis sample presented more than twice the number of HLA-B*07 phosphopeptides, indicating that this increased presentation is actually driven by the metastatic status of the tumor. We show that at least a quarter of these HLA-I phosphopeptides are derived from essential cancer signaling pathways, which may therefore limit the scope for tumor immune escape by loss of antigen (**Supplementary Figure 5**) (42). Since little is known about the effects of phosphorylation at the specific sites on most of the phosphopeptide source proteins (**Supplementary Tables 3**–**5**), it is difficult to reach firm conclusions about their contribution to the malignant state, creating the possibility that even more CRC phosphopeptides are implicated in cancer signaling than currently described.

Secondly, we have shown that these HLA-I phosphopeptides are public antigens, with up to 40% of the phosphopeptides presented by common HLA alleles found on more than one sample in our small cohort (**Figure 2C** and **Supplementary Figure 2C**). This would make any targeted therapies widely applicable at a population level, potentially against any malignancy. Of note, many of the phosphopeptides identified were attributed to HLA-C*07, a common allele (37-69%) across European and African populations and known to contribute significantly to tumor immunity (43, 44). Further examination of a larger CRC patient cohort is needed to confirm which phosphopeptides are most frequently presented. Promisingly, some of the identified phosphopeptides were predicted to bind different HLA-I alleles in different patients, suggesting that presentation may be not be limited by specific HLA-type, but apply across different HLA superfamilies (**Supplementary Tables 3**–**5**) (41).

Thirdly, we have shown that some of the tumor-resident immunity in CRC, which is known to be of prognostic significance, targets HLA-I phosphopeptides (**Figures 3** and **4**) (1, 3, 5, 9). This was true of TILs in the tumors in which we had identified the phosphopeptides (**Figure 3**), and in a further cohort of primary CRC tumors (**Figure 4A**). Previously, it has been suggested that many of the TILs in CRC are merely bystanders - not directly targeting the tumor (45). Indeed, viral responses were also assayed here, and these bystander responses were strong. However, we clearly demonstrate that at least some TILs are targeting phosphopeptides. The frequency of TILs targeting each individual phosphopeptide was low, but this has also been seen with other tumor antigen specific TILs (46). Taken together with the data from patient PBMCs, we can conclude that HLA-I phosphopeptide-specific TILs represent a subset of the tumor-resident immunity to CRC. We have also shown that phosphopeptide-specific T cells can target and kill tumor cells (**Figures 5C, D** and **Supplementary Figure 13**). Thus, these may prove ideal candidates for future immunotherapeutic strategies.

The use of phosphopeptides as immunotherapeutic targets would have limitations. Primarily, the presence of HLA-I phosphopeptides on other healthy tissues, which we discovered particularly on the “healthy” liver tissue, neighboring CRC liver metastases. This could be due to the presence of microscopic metastases; however, it may also be attributable to the role of the liver in tolerizing antigen from the gastrointestinal tract (47). There were TILs that targeted HLA-I phosphopeptides identified on healthy tissue. If this were not simply due to micrometastases, it might suggest a low T cell functional avidity, which would prevent auto-reactivity and enable targeting of tumor cells, where antigen density is much higher (48). Therefore, in these cases it would not be appropriate to use any therapeutic strategies that involve the modulation of TCR affinity, such as bispecifics (39). Strikingly, peripheral T cell responses were detected in heathy donors targeting most of the CRC phosphopeptides (**Figures 4B, C**). These responses may be initiated in healthy donors when pre-malignant transformation events occur, perhaps caused by infection with transforming viruses (19). In CRC, transforming events may also be initiated by bacteria, such as enterotoxigenic *Bacteroides fragilis* or *Fusobacterium nucleatum* (49, 50). The fact that some healthy donors have T cells targeting these phosphopeptides suggests that these phosphopeptides are not presented on other healthy tissues, since these donors are not showing symptoms of autoimmunity. Furthermore, phase I clinical trials using a phosphopeptide vaccine have proven safe in melanoma, which included one of the CRC phosphopeptides of interest RVAsPTSGV (40). Therefore, any therapy targeting these PTM tumor antigens should not be limited by off-target effects.

Another potential limitation of therapeutically targeting HLA-I phosphopeptide antigens is the lack of functional response seen in patient TILs *in vitro*. We show that phosphopeptide-specific T cell lines can kill CRC cell lines in a phosphopeptide-specific manner (**Figures 5C, D** and **Supplementary Figure 13**). This is in accordance with data from previous studies, where phosphopeptide-specific T cells were observed to kill *ex vivo* from leukemia patients and *in vivo* in a mouse melanoma model (19, 51). However, a line derived from CRC patient TILs, although activated, did not kill (**Figures 5A, B**). This difference in function may explain why the tumor is persisting in these patients - perhaps these T cells have become exhausted and no longer kill, or they may be functionally altered by the tumor microenvironment (52, 53). Recent studies in CRC have suggested roles for immune checkpoints other than PD-1, and exhaustion markers, such as CD39, CD73, TIM-3, LAG-3 and TIGIT (52). Limitations due to exhaustion or checkpoint blockade could be overcome using combination therapies with a checkpoint blockade inhibitor, or therapies targeting coinhibitory receptor molecules, or enhancing costimulatory immune checkpoint molecules, as has proven effective in preclinical vaccine models (54). An alternative explanation could be that the TILs lost function during the expansion *in vitro* (55), since using “young” TILs we observed phosphopeptide-specific upregulation of degranulation marker CD107a (**Figure S14**). These *in vitro* effects could be overcome using modified expansion protocols, to provide successful ATC therapies (56).

This study is too small to establish the clinical significance of patient T cell responses targeting HLA-I phosphopeptides. Previously, we observed that CLL patients without immunity to phosphopeptides did less well clinically (19). Although anecdotal at this stage, here we saw very few TILs targeting HLA-I phosphopeptides in the one CRC patient (CRC6) who did suffer recurrence (**Figure 4A**). A larger scale study in CRC is needed to determine whether having higher responses targeting tumor-specific phosphopeptides correlates with better patient outcomes. Intriguingly, we observed stronger peripheral T cell responses targeting many of the HLA-I phosphopeptides in CRC patients than healthy donors, whereas previously, in leukemia, we showed that a subset of patients have absent or impaired responses to phosphopeptides (19). The difference between the PBMC responses in CRC and leukemia patients may be due to the inherent difference between solid tumors and blood malignancies – that the tumor microenvironment can be controlled in a solid tumor, so whilst CRC patients may retain phosphopeptide-specific T cells their function may be impaired at the tumor site. Although there were also HLA-I phosphopeptides that did not elicit T cell responses from CRC patients (**Figures 4B, C**), given that CD8 T cells are known to control tumor growth in CRC, then the more interesting responses may be those that we have seen in tumor-resident T cells and observed to be higher in CRC patients’ peripheral blood (1–3, 9). This approach is further supported by recent reports describing the presence of memory T cells in the peripheral blood of CRC patients that target tumor-associated antigens (57), and mutational neoantigens (58). Using T cells extracted from the peripheral blood in ATC therapies would have demonstrable benefits over the requirement for surgically obtained TILs.

In conclusion, we have shown that the targets of tumor-resident CD8 T cells include PTM tumor antigens, namely HLA-I phosphopeptides. Once these findings have been validated in a larger patient cohort, tumor-specific phosphopeptides may provide an optimal target for future immunotherapeutics. Critically, these HLA-I associated phosphopeptides are public antigens - often shared across patients presenting the same HLA molecules. This would make any targeted therapies widely applicable at a population level, potentially against any malignancy. Our future efforts will be devoted to expanding this work into larger patient cohorts to further our understanding of phosphopeptide targeting by effector T cells, and to ultimately develop more efficacious immunotherapies for CRC.

## Data Availability Statement

The original contributions presented in the study are included in the article/**Supplementary Material**. Further inquiries can be directed to the corresponding author.

## Ethics Statement

The studies involving human participants were reviewed and approved by ethics committees local to the University of Birmingham (Ref 09/H1010/75, 06/Q2702/61 and 09/H1203/49). The patients/participants provided their written informed consent to participate in this study.

## Author Contributions

SP was involved in the conception and design of the study, acquisition and analysis of data and wrote manuscript. JA designed and performed MS experiments, analysed data, and participated in manuscript writing. SM participated in manuscript writing. AS performed scientific experiments. PM performed MS experiment and analysed data. LS performed scientific experiments. SW assisted with sample collection. DB wrote in-house software and analysed MS data. DH and JS directed MS experiments and analysed MS data. MC was involved in conception of the work, directed experiments and participated in manuscript writing. All authors contributed to the article and approved the submitted version.

## Funding

This work was supported by Queen Elizabeth Hospital Birmingham Charity grant RCDE13729 (to MC), NIH-AI033993 (to DH).

## Conflict of Interest

MC owns equity in Revitope Oncology and Gritstone Oncology and now is an employee of AstraZeneca. JS and DH own stock in Agenus. The discovery here makes up patent Target peptides for colorectal cancer therapy and diagnostics, WO2014/039675. 2014 Mar 13 with inventors MC, SP, DH, JS and JGA.

The remaining authors declare that the research was conducted in the absence of any commercial or financial relationships that could be construed as a potential conflict of interest.

## Publisher’s Note

All claims expressed in this article are solely those of the authors and do not necessarily represent those of their affiliated organizations, or those of the publisher, the editors and the reviewers. Any product that may be evaluated in this article, or claim that may be made by its manufacturer, is not guaranteed or endorsed by the publisher.
