# Supplementary material for: Tumor Infiltrating Lymphocytes Target HLA-I Phosphopeptides Derived From Cancer Signaling in Colorectal Cancer
Source: Front Immunol. 2021 Aug 24;12:723566. doi: 10.3389/fimmu.2021.723566 (PMC8421858; doi:10.3389/fimmu.2021.723566)
Supplement: Supplementary file 1 [file DataSheet_1.pdf]

	Patient age	Tumor position	Tumor size (mm)	Histological type	Tumour Grade	Nodal Status	Met-astases	Chemo-therapy	Recurrence	Other	HLA type		
CRC1	82	anterior resection	110	Adenocarcinoma of rectum	3	0	x	-	N/A		A*03, A*29	B*07, B*51	C*02, C*07
CRC2	84	left hemicolectomy	115 x 120 x 80	Adenocarcinoma	3	0	x	-	-		A*02, A*23	B*44	C*04, C*05
CRC3	57	right hemicolectomy	70 x 60	Adenocarcinoma of caecum	2B	0	x	neoadjuvant 5FU and cisplatin	-		A*02, A*31	B*40, B*45	C*03, C*06
CRC4	78	right hemicolectomy	45 x 30 x 20	Adenocarcinoma of caecum	4	1	x	adjuvant Capecitabine and Oxaliplatin	-		A*02, A*03	B*07, B*14:02	C*07, C*08
CRC5	73	right hemicolectomy	80 x 130 x 35	Adenocarcinoma of caecum	4	1	x	adjuvant Capecitabine and Oxaliplatin	-	K-RAS mutation	A*02	B*35, B*50:01	C*04, C*06
CRC6	61	left hemicolectomy	65 x 40 x 13	Adenocarcinoma of sigmoid	3	0	x	-	+		A*02, A*03	B*35, B*58	C*04, C*07

SUPPLEMENTARY TABLE S1 | Clinical data from patients of primary CRC samples used

Patient samples were obtained at colorectal cancer resection. This table shows the clinical data for the three primary colorectal cancer specimens used in phosphopeptide discovery and the six used for extraction of tumor infiltrating lymphocytes for immunological assays.

	Tumor sample	Healthy tissue	Patient age	Primary tumor			Time to progression	Other malignancies	HLA type		
				Time since resection	Position	Dukes stage					
**CRCLM1**	Liver metastasis	Liver	69	5 and 2 years	Rectal	B	6 weeks	CLL	A*02, A*03	B*07	C*07
**CRCLM2**	Liver metastasis	Liver	70	1 year	Rectal	B	N/A	-	A*02, A*24	B*08, B*27	C*07

SUPPLEMENTARY TABLE S2 | Clinical data from patients of CRC liver metastasis samples used

Patient samples were obtained at resection of CRC liver metastases. This table shows the clinical data for the two colorectal cancer liver metastases used in phosphopeptide discovery and for extraction of tumor infiltrating lymphocytes for immunological assays.

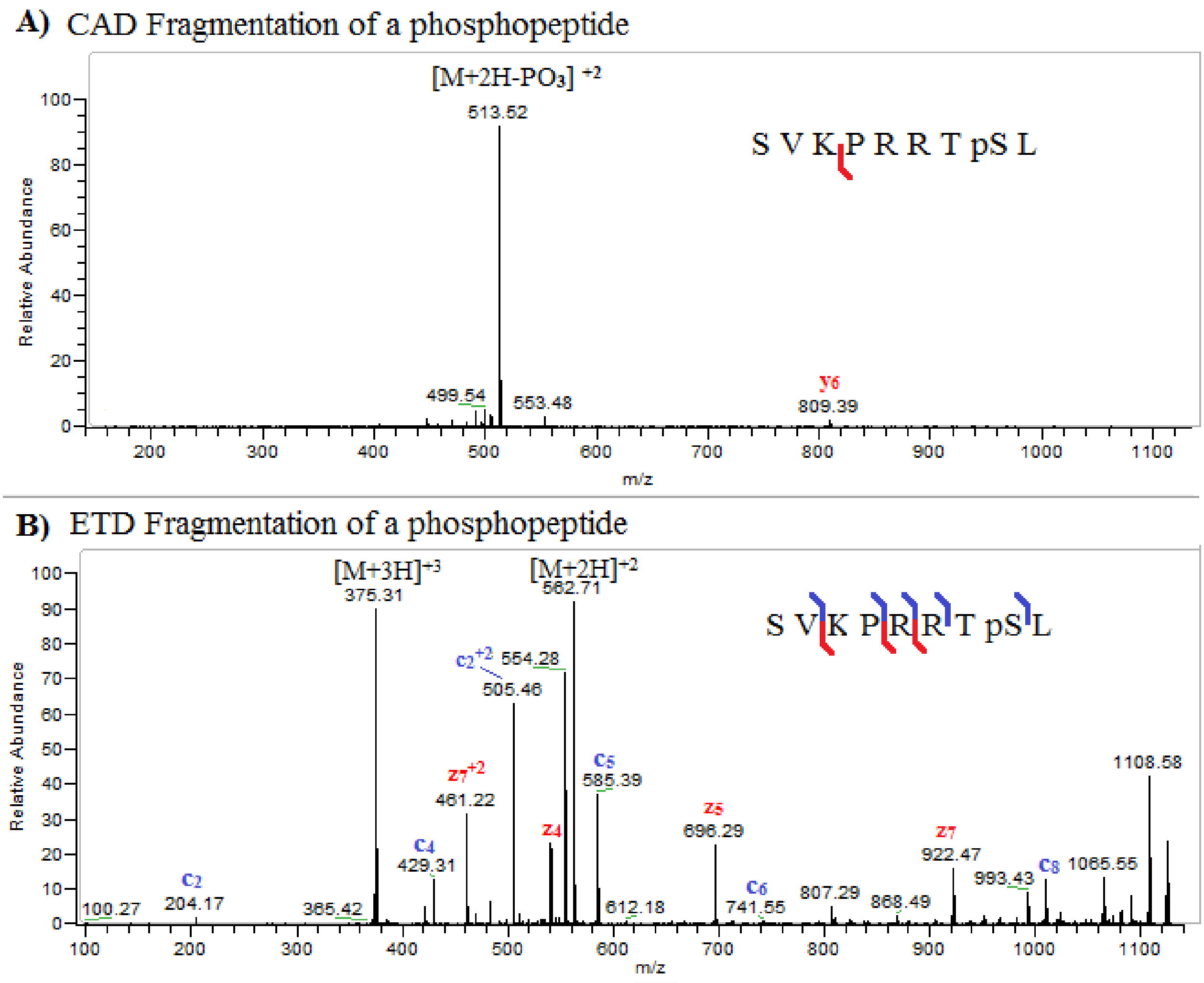

SUPPLEMENTARY FIGURE S1 | Fragmentation of phosphopeptides

(A) CAD fragmentation results in the domination of a spectrum by the neutral loss of phosphoric acid, which can be used as a diagnostic in "CAD Neutral Loss Finder." (B) ETD fragmentation preserves the post-translational modification, which allows us to sequence the phosphopeptide.

HLA binding	Phosphopeptide	Uniprot #	Protein Name	CRC1 Healthy	CRC1 Tumour	CRC2 Healthy	CRC2 Tumour	CRC3 Healthy	CRC3 Tumour	LM1 Healthy	LM1 Tumour	LM2 Healthy	LM2 Tumour	colo205	sw620	hct116
A*01	ITQGtPLKY	Q9Y618	NCOR2											10.00-99.99		1.00-9.99
	ISSsMHSLY	P50616	TOB1											1.00-9.99		
	LTDPSsPTISSY	Q8IX90	SKA3											1.00-9.99		
	NTDsPLRY	P08865	RPSAP19													10.00-99.99
	TMAsPGKDNY	O60684	KPNA6											1.00-9.99		1.00-9.99
A*02	RQIsQDVKL	Q01433	AMPD2				1.00-9.99								0.10-0.99	
	RVAsPTSGV	Q9Y4H2	IRS2											1.00-9.99		1.00-9.99
	RQAsIELPSMAV	P33241	LSP1						10.00-99.99							
	ALDsGASLLHL	P57078	RIPK4								0.10-0.99					
	RLAsYLDRV	P05783	KRT18P19										0.10-0.99			
	KLIDRTEsL	P33241	LSP1										0.10-0.99			
	RIsHELDS	P10451	SPP1										0.10-0.99			
	LLLsEEVEL	Q8IY92	BTBD12											1.00-9.99		
	VMIGsPKKV	Q68CZ2	TNS3												10.00-99.99	
	SMTRsPPRV	Q9BRL6	SFRS2B												1.00-9.99	
	KLAsPELERL	P05412	JUN												1.00-9.99	
	KLIDIVsSQKV	O14757	CHEK1												1.00-9.99	
	FLDtPIAKV	Q969G9	NKD1												1.00-9.99	
	RTLsPEIITV	Q9H4A3	WNK1												1.00-9.99	
	RVLHsPPAV	Q9Y4B5	MTCL1												1.00-9.99	
	RLSsPLHFV	Q8NC44	FAM134A												0.10-0.99	
	VmIGsPKKV	Q68CZ2	TNS3												0.10-0.99	
	RTHsLLLLL	P34096	RNASE4			10.00-99.99		10.00-99.99		0.10-0.99	10.00-99.99	1.00-9.99			1.00-9.99	
	RQAsIELPSM	P33241	LSP1			1.00-9.99	1.00-9.99		10.00-99.99				1.00-9.99			
A*03	RIYQyIQSR	Q9Y463	DYRK1B		1.00-9.99											
	RILsGVVTK	P62280	RPS11		0.10-0.99											
	GImsPLAKK	Q03989	ARID5A		0.10-0.99											
	RLSsPISKR	Q99728	BARD1								1.00-9.99					
	RVAsPTSGVK	Q9Y4H2	IRS2								1.00-9.99					
	RTRsLSSLREK	O94915	FRYL								1.00-9.99					
	RTMsEAALVRK	Q6ZTQ3	RASSF6								0.10-0.99					
	SVRRsVLmK	Q9H2J4	PDCL3								0.10-0.99					
	KLPsPAPARK	Q8IY33	MICALL2							0.10-0.99	1.00-9.99					
	RVLsPLIIK	Q8NCN4	RNF169		0.10-0.99					1.00-9.99						
A*24	RFKtQPVTF	Q7Z7L8	AG2			1.00-9.99	1.00-9.99						0.10-0.99			
	RYQtQPVTL	O95425	SVIL												1.00-9.99	
A*29	RIYQyIQSRF	Q9Y463	DYRK1B		0.10-0.99											

Key (fmol/g tissue)

- > 100.00
- 10.00-99.99
- 1.00-9.99
- 0.10-0.99
- Not detected

SUPPLEMENTARY TABLE S3 | Phosphopeptides identified on CRC patient samples that are predicted to bind to HLA-A

MHC class I-bound peptides were eluted from tumor tissue and it's healthy counterpart, or cultured cell lines. Phosphopeptides were IMAC enriched and characterized using LC-MS/MS. MHC class-I binding was predicted, using known HLA-alleles. Here 3 primary CRC, 2 CRC liver metastases and 3 cell line samples were analyzed.

HLA binding	Phosphopeptide	Uniprot #	Protein Name	CRC1 Healthy	CRC1 Tumour	CRC2 Healthy	CRC2 Tumour	CRC3 Healthy	CRC3 Tumour	LM1 Healthy	LM1 Tumour	LM2 Healthy	LM2 Tumour	colo205	sw620	hct116
B*07	LPIFSRLsI	P47974	ZFP36L2		1.00-9.99						1.00-9.99					
	RPKtPPVVI	Q96A49	SYAP1		0.10-0.99						1.00-9.99					
	SPFKRQLsL	B7Z5W0	NUMBL		1.00-9.99					1.00-9.99	10.00-99.99					
	RPRsPNMQDL	Q6T310	RASL11A		1.00-9.99											
	LPVsPRLQL	P13688	CEACAM1		0.10-0.99											
	AVRPTRLsL	Q9Y4H2	IRS2								10.00-99.99					
	SPRsPDRTL	Q9UKN1	MUC12								10.00-99.99					
	QPQRRsLRL	Q9ULW0	TPX2								1.00-9.99					
	VPRPERRsSL	Q6UWJ1	TMCO3								1.00-9.99					
	KPEsRRSSLL	Q6WKZ4	RAB11FIP1								1.00-9.99					
	APRKGsFSAL	Q13619	CUL4A								1.00-9.99					
	RPTKIGRRsL	Q96HN2	AHCYL2								1.00-9.99					
	RPDVAKRLsL	O75815	BCAR3								1.00-9.99					
	RPFHGISTVsL	Q5VZ89	DENND4C								1.00-9.99					
	RPVtPVSDL	Q13118	KLF10								1.00-9.99					
	RPGsRQAGL	Q96JY6	PDLIM2								1.00-9.99					
	RPDsAHKML	Q8WX93	PALLD								1.00-9.99					
	RPRARsVDAL	Q86X29	LSR							10.00-99.99	> 100.00					
	RARGIsPIVF	Q96MU7	YTHDC1							0.10-0.99	1.00-9.99					
	APDsPRAFL	unknown	UNKNOWN							0.10-0.99	1.00-9.99					
	SPRSPsTTYL or SPR	Q13111	CHAF1A											1.00-9.99		
	RPFsPREAL	Q86V48	LUZP1		1.00-9.99					10.00-99.99	10.00-99.99					
	RPRPVsPSSL	P57059	SIK1		1.00-9.99					1.00-9.99	1.00-9.99					
	KPAsPKFIVTL	Q6PJT7	ZC3H14		0.10-0.99					1.00-9.99	1.00-9.99					
	TPRsPPLGL	Q16584	MAP3K11		0.10-0.99					1.00-9.99	1.00-9.99					
	RPRsPTGPSNSF	Q96I25	RBM17		0.10-0.99					1.00-9.99						
	RPVsPFQEL	unknown	UNKNOWN		0.10-0.99					1.00-9.99	1.00-9.99					
	KPRsPVVEL	P25098	ADRBK1	10.00-99.99	10.00-99.99					> 100.00	10.00-99.99			10.00-99.99		
	RPRGsQSLL	P21860	ERBB3							10.00-99.99	10.00-99.99			10.00-99.99		
	RPAsPQRAQL	unknown	UNKNOWN							10.00-99.99	10.00-99.99			10.00-99.99		
B*08	DLKRRsMSI	Q96N67	DOCK7										0.10-0.99			
	SVKPRRTsL	P15822	HIVEP1											1.00-9.99		
	INKERRSsL	Q5JTZ5	C9ORF152											1.00-9.99		
B*15	RQDsTPGKVFL or F	P13056	NR2C1												1.00-9.99	
B*27	GRLsPAYSL	Q86UU1	PHLDB1										1.00-9.99			
	GRLsPVPVPR	Q9UKM9	RALY										0.10-0.99			
	RRFsRLENRY	O43293	DAPK3										0.10-0.99			
	RRDsLQKPGL	Q9NRM7	LATS2										0.10-0.99			
	RRAsQEANL	Q6PJG2	C14ORF43										0.10-0.99			
	RRLsLFLNV	Q99836	MYD88										0.10-0.99			
B*27/C*07	KRFsFKKsF	P29966	MARCKS										1.00-9.99			
	RRLsDSPVF	P47974	ZFP36L2										1.00-9.99			
	KRYsGNMEY	O95835	LATS1										1.00-9.99			
	RRMsLLSVV	Q9ULI2	RIMKLB										0.10-0.99			
	RRSsFLQVF	Q15436	SEC23A										0.10-0.99			
	RRSsIQSTF or RRsS	Q92542	NCSTN										0.10-0.99			
	RRLsESSAL	Q96S55	WRNIP1										0.10-0.99			
	RRNsINRNF	O00160	MYO1F									0.10-0.99	1.00-9.99			
B*44	AENsPTRQQF	Q86XP3	DDX42				1.00-9.99									
	KEMsPTRQL	Q4G0N7	C6ORF225				1.00-9.99									
	AIsDLQQL	O15302	CAMK2				1.00-9.99									
B*45	EERRsPPAP	P15408	FOSL2					10.00-99.99	> 100.00							10.00-99.99
	EERsPSWISA	Q02952	AKAP12													> 100.00
	EER**sts**WISA	Q02952	AKAP12													> 100.00
	EERsPSwISA	Q02952	AKAP12													> 100.00
	REIsSSPTS	Q9UQ35	SRRM2													1.00-9.99
	AEAPPSKsP	Q96D71	REPS1													1.00-9.99
	AEKsYQNSP	Q15648	MED1													1.00-9.99
	SEAsPSREA	Q13111	CHAF1A													0.10-0.99
B*51	FEDDDsNEKL	O43719	HTATSF1		0.10-0.99											

Key

- \> 100.00 fmol/g tissue
- 10.00-99.99 fmol/g tissue
- 1.00-9.99 fmol/g tissue
- 0.10-0.99 fmol/g tissue
- Not detected

SUPPLEMENTARY TABLE S4 | Phosphopeptides identified on CRC patient samples that are predicted to bind to HLA-B

HLA binding	Phosphopeptide	Uniprot #	Protein Name	CRC1		CRC2		CRC3		LM1		LM2		colo205	sw620	hct116
				Healthy	Tumour	Healthy	Tumour	Healthy	Tumour	Healthy	Tumour	Healthy	Tumour			
C*03	KAFsPVRSV	Q02363	ID2						10.00-99.99						1.00-9.99	
	RAHSsPASL	P46937	YAP1					1.00-9.99	10.00-99.99							
	RSHSsPASL	Q9GZV5	WWTR1					1.00-9.99	10.00-99.99							
C*05	RADsPVHM	O95402	MED26				0.10-0.99									
	RRDsIVAEL	O14579	COPE				0.10-0.99					1.00-9.99	0.10-0.99			
	SIDsPQKL	Q12888	TP53BP1													10.00-99.99
	RSDsYVEL	P52298	NCBP2													1.00-9.99
C*06	RRSsSVAQV	O15205	UBD						10.00-99.99							
	RRPsLLSEF	O75376	NCOR1						10.00-99.99							
	RRNsAPVSV	Q2M1Z3	ARHGAP31						10.00-99.99							
C*07	RRGsFEVTL	Q8IZQ5	SELH		1.00-9.99					1.00-9.99	10.00-99.99	1.00-9.99	1.00-9.99			
	RRLsFLVSY	P47897	QARS		0.10-0.99								0.10-0.99			
	RKLsVILIL	Q13433	SLC39A6		0.10-0.99											
	KRFsGTVRL	P62906	RPL10AP9								1.00-9.99					
	RRSsQSWSL	Q9Y4E1	FAM21C								1.00-9.99					
	HRNsMKVFL	Q9NPR2	SEMA4B										0.10-0.99			
	RRKsQVAEL	Q9BYG3	MKI67IP							1.00-9.99	10.00-99.99					
	KRLsVERIY	P11388	TOP2A											1.00-9.99		
	RRLsGPLHTL	Q86Y91	KIF18B											1.00-9.99		
	KLFPDtPLAL	Q12906	ILF3												1.00-9.99	
	KYIsGPHEL	P49454	CENPF												0.10-0.99	
	RRFsGTAVY	Q6AHZ1	ZNF518A		0.10-0.99					1.00-9.99	1.00-9.99	0.10-0.99	0.10-0.99			
	MPRQPsATRL	Q6NZ67	FAM128B							10.00-99.99	10.00-99.99			1.00-9.99		
C*07/B*27	HRLsPVKGEF	Q9Y2L9	LRCH1										0.10-0.99			
	RRIDIsPSTLR	Q9NYF8	BCLAF1										0.10-0.99			
	RRFsPPRRM	Q15287	LOC643446										0.10-0.99			
	RRIsGVDRYY	O15239	NDUFA1										0.10-0.99			
	KRMsPKPEL	P41208	CETN2									1.00-9.99	1.00-9.99	1.00-9.99		
C*06/C*07	RRIsDPQVF	Q4L180	FILIP1L	10.00-99.99	1.00-9.99			10.00-99.99	> 100.00	1.00-9.99	10.00-99.99		1.00-9.99			

Key (fmol/g tissue)
- > 100.00
- 10.00-99.99
- 1.00-9.99
- 0.10-0.99
- Not detected

SUPPLEMENTARY TABLE S5 | Phosphopeptides identified on CRC patient samples that are predicted to bind to HLA-C

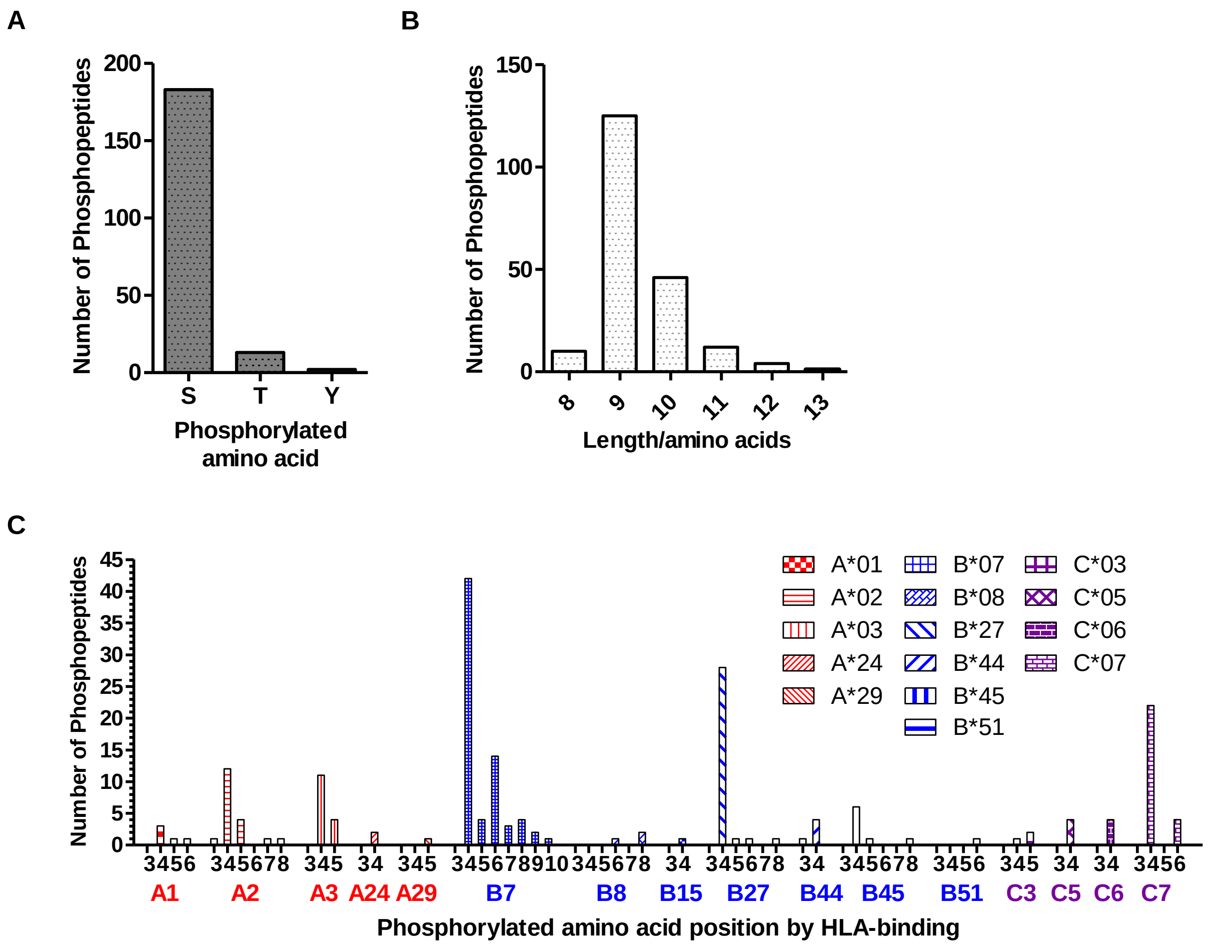

SUPPLEMENTARY FIGURE S2 | Phosphopeptide characteristics

(A) The number of CRC phosphopeptides identified containing phospho-serine (S), phospho-threonine (T) and phospho-tyrosine (Y).
(B) The lengths of the CRC phosphopeptides identified.
(C) The position of the phosphorylated amino acid in the phosphopeptides, according to HLA-binding.

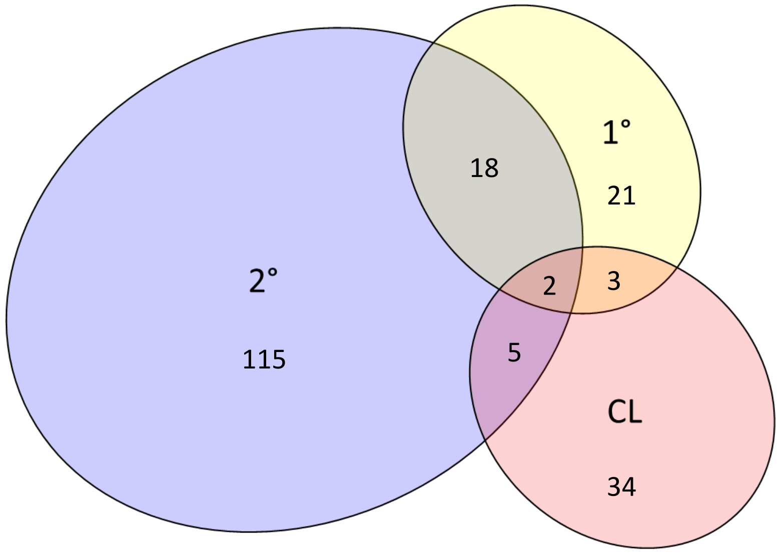

SUPPLEMENTARY FIGURE S3 | How the phosphopeptides identified are shared between samples

A Euler plot showing the overlap of presentation of phosphopeptides between differing types of CRC samples: - primary CRC – 1°; secondary CRC liver metastases – 2°; and cell lines –CL.

A

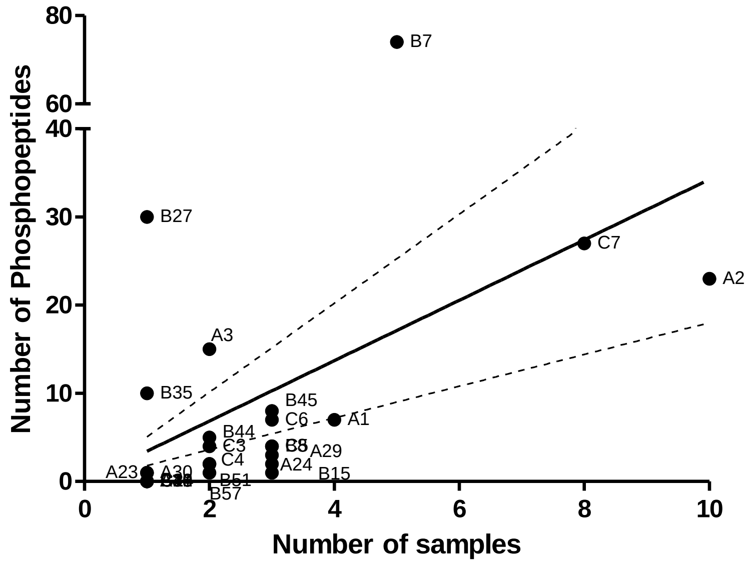

B

Kinase name	Consensus sequence	# phosph. sites
PKACa (PRKACA)	xxrxRRlSlxxxxxx	734
PKCa (PRKCA)	xxxxRRxSfKrkkxx	523
CK2a1 (CSNK2A1)	xxxeeedSDdEeeee	483
ERK2 (MAPK1)	xxpxpPlSPtppxxx	410
CDK1 (CDC2)	xxxxlpxSPxkkxxx	393
SRC	xxxeedvYgxvxxxx	385
ERK1	xxpppPlSPtptxxx	292
CDK2	xxxxxpxSPgKkxlx	201
PDK1 (PDPK1)	xxgxttxTFCGTpeY	43

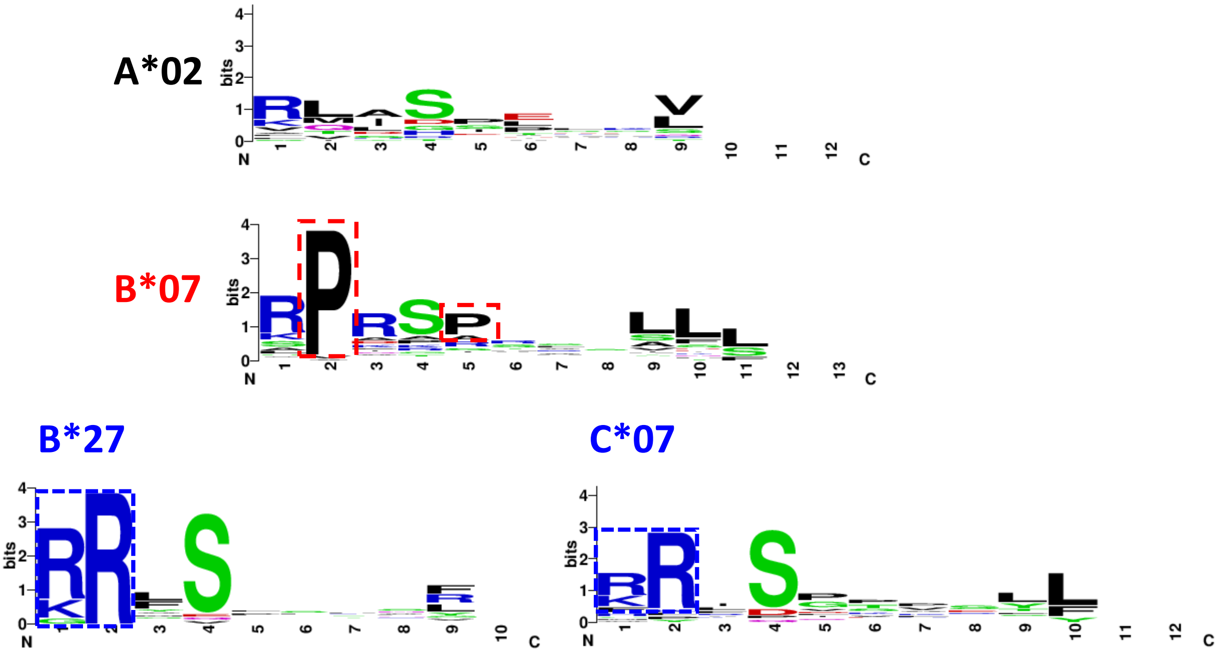

SUPPLEMENTARY FIGURE S4 | Phosphopeptides binding motifs vs. kinase binding motifs

(A) The number of phosphopeptides identified, plotted against the number of samples of each HLA-type. HLA-B*07 and HLA-B*27 are shown to be outliers, with many more phosphopeptides per sample than the general trend.

(B) Published kinase binding motifs are compared to logoplots of the phosphopeptide sequences identified on CRC, showing key consensus sequences around the phosphoserine are shared for HLA-B*07 with ERK1 and ERK2; and for HLA-B*27 and HLA-C*07 with PKACa and PKCa.

	Phospho-peptide	Uniprot #	Source protein	Malignant Samples		
				CRC	Leukemia	Melanoma
A*02	RVAsPTSGV	Q9Y4H2	IRS2	colo205, hct116	CLL1, CLL2, MCL	DM331, SLM2, COV413
	VMIGsPKKV	Q68CZ2	TNS3	sw620	MCL	DM331, SLM2, COV413
	VmIGsPKKV	Q68CZ2	TNS3	sw620	MCL	DM331, SLM2, COV413
B*07	GPRSAsLLSL	Q9Y4H4	GPSM3	CRCLM1	AML1	
	RPFsPREAL	Q86V48	LUZP1	CRC1, CRCLM1	AML1, ALL1, CLL2, CLL4,HCL1, B-LCL	
	RPRPVsPSSL	P57059	SIK1	CRC1, CRCLM1	AML1	
	RPRsPRQNSI	Q99700	ATXN2	CRCLM1	AML1, B-LCL	
	RPVsPFQEL	unknown	unknown	CRC1, CRCLM1	AML1, ALL1, CLL2, CLL4,HCL1, B-LCL	
	TPRsPPLGL	Q16584	MAP3K11	CRC1, CRCLM1	AML1, CLL2, CLL4,HCL1, B-LCL	

SUPPLEMENTARY TABLE S6 | Phosphopeptides shared across different malignancies have been identified in multiple samples

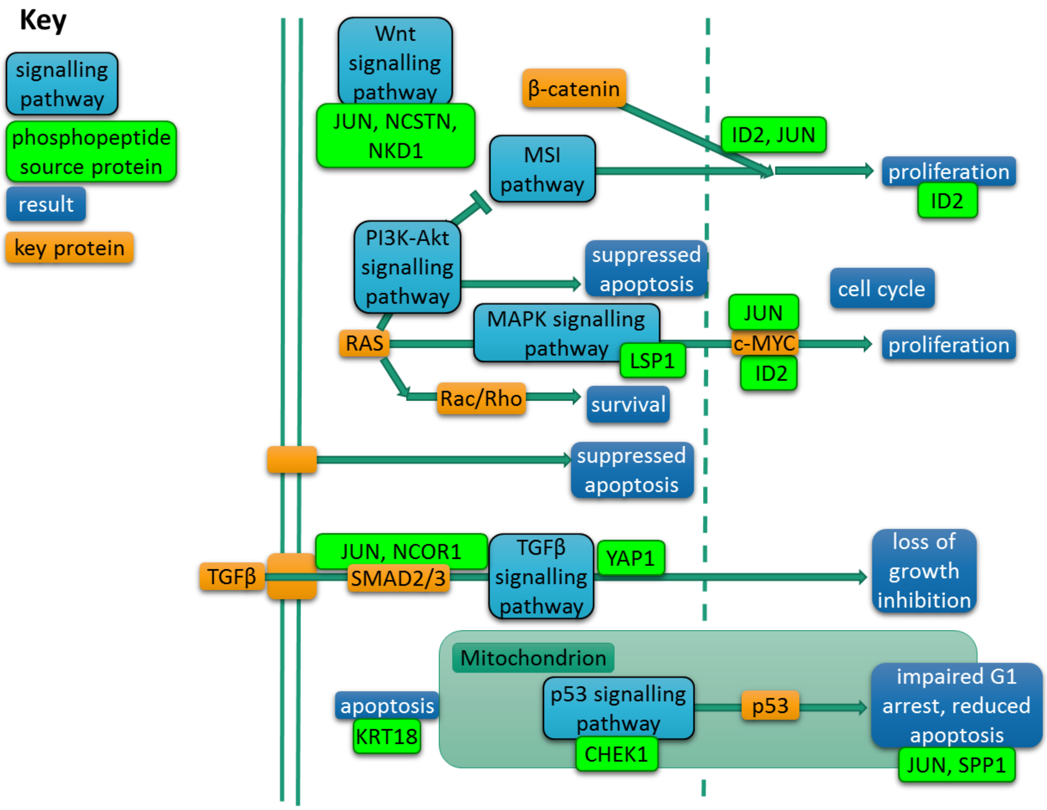

SUPPLEMENTARY FIGURE S5 | The source proteins of phosphopeptides identified superimposed onto the KEGG CRC signaling pathways

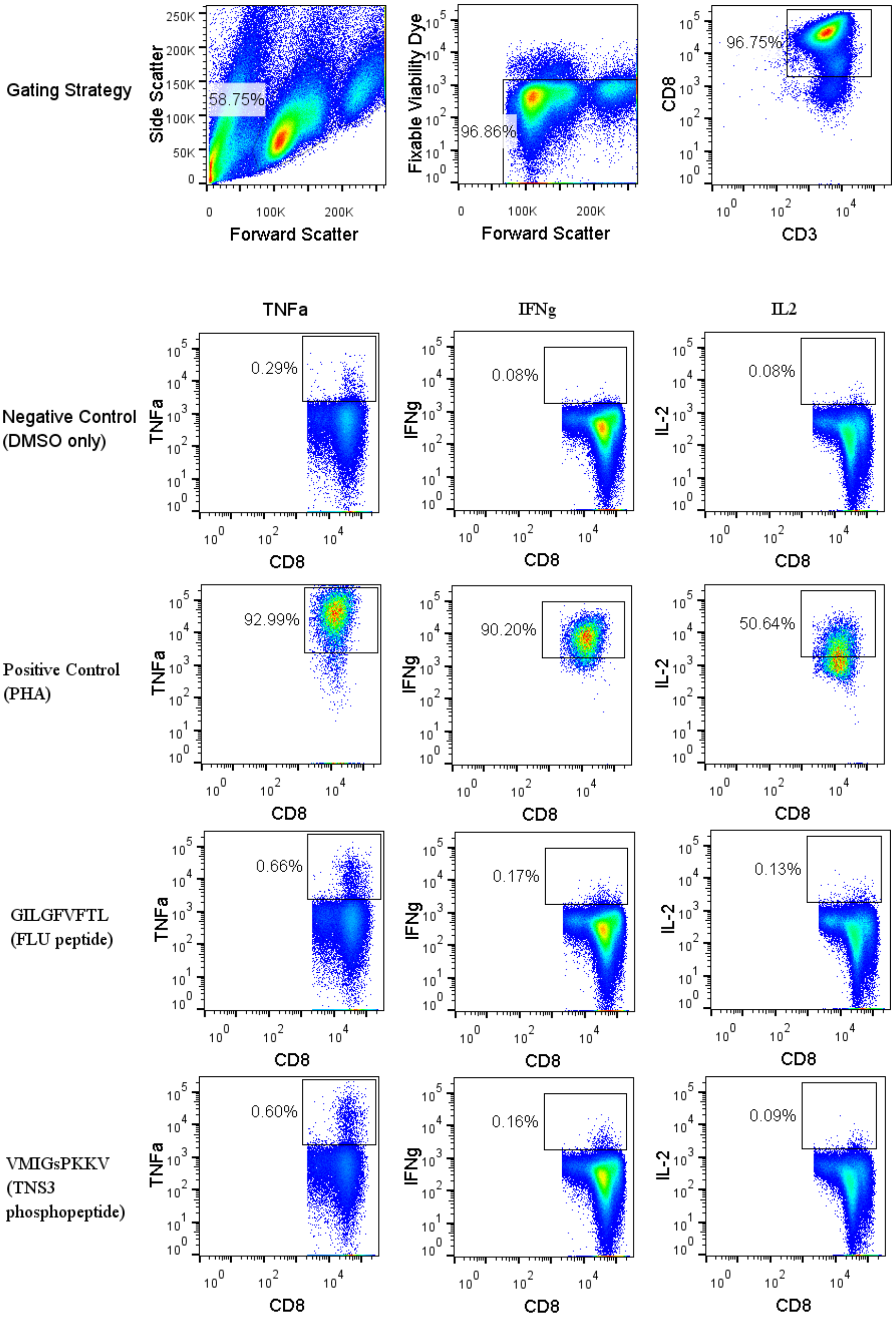

SUPPLEMENTARY FIGURE S6 | Batch analysis of Intracellular Cytokine Staining (ICS) used to assess TIL targeting of phosphopeptides

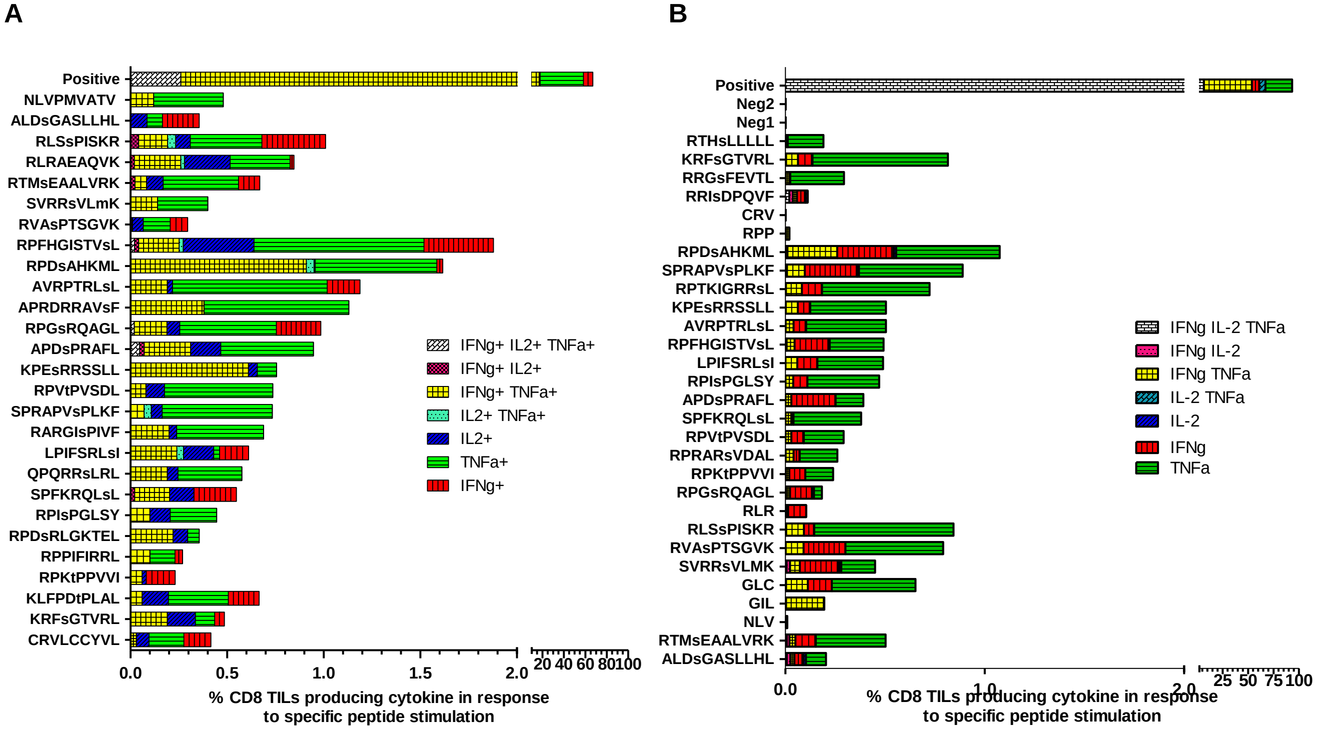

SUPPLEMENTARY FIGURE S7 | Raw data for the two different CRCLM1 ICS assay repeats

(A) After one REP cycle of TILS and (B) after a second REP cycle.

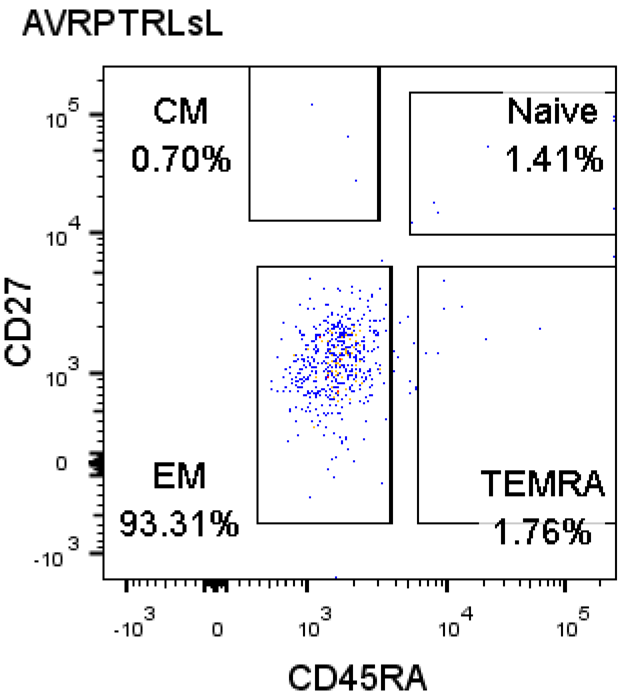

SUPPLEMENTARY FIGURE S8 | An example of memory phenotype of TIL cultures targeting CRC-associated phosphopeptides

CD45RA and CD27 staining was used to gate central memory (CM), effector memory (EM), naïve and terminal effector memory (TEMRA) phenotypes.

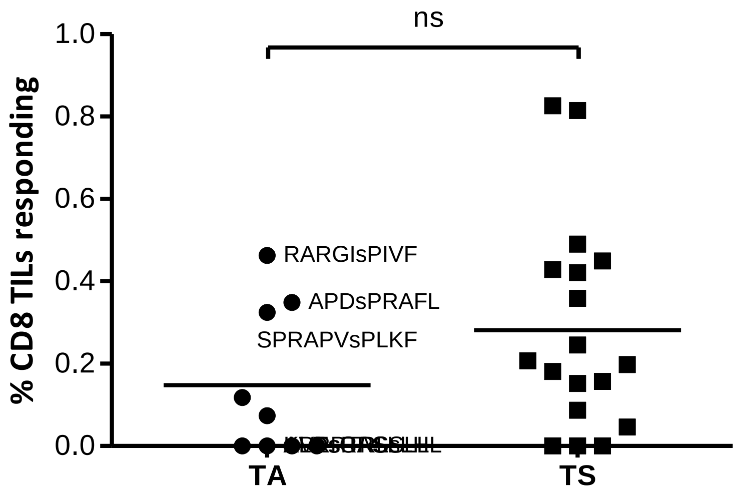

SUPPLEMENTARY FIGURE S9 | CRCLM1 TIL responses targeting phosphopeptides grouped by presentation on tumor and healthy tissue

TIL responses targeting CRC phosphopeptides that were present on both healthy and tumor tissue - TA (tumor-associated) and those that were present only on tumor tissue - TS (tumor-specific).

CRC #	Patient age (at time of sample)	Histology	Tumour size (mm)	Surgery type	Tumour Grade	Nodal Status	Metastases	Neo-Adjuvant RT
22	68	Adenocarcinoma	20 x 25 x 10	Right hemicolectomy	1	0	X	NIL
23	74	Adenocarcinoma	40 x 83 x 15	Right hemicolectomy	3	0	x	NIL
25	48	Mucinous adenocarcinoma	65 x 85 x 12	Subtotal colectomy	3	0	x	NIL
26	74	Adenocarcinoma of rectum	30 x 20 x 6	Anastamotic doughnut	3	0	x	25 Gray in 5 fractions
27	66	Adenocarcinoma	60 x 50	Left colon distal doughnut	2	0	x	NIL

SUPPLEMENTARY TABLE S7 | Clinical data of CRC patients whose PBMCs were used in the ELISpot assay

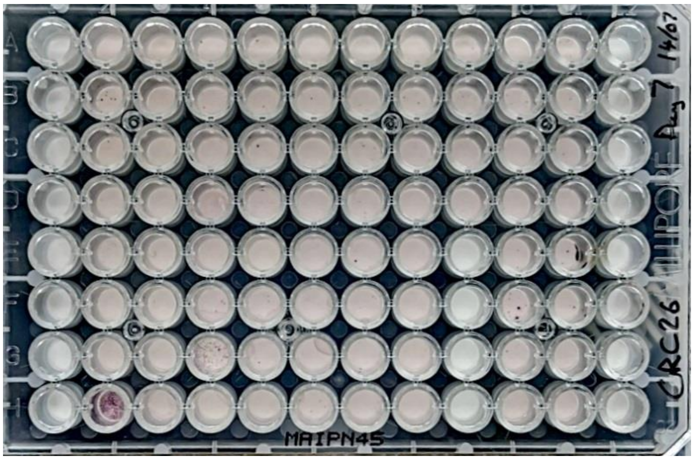

SUPPLEMENTARY FIGURE S10 | IFNγ ELISpot was used to assess *ex vivo* T cell responses to phosphopeptides from healthy donor and CRC patient PBMCs

Blood was collected from healthy donors and CRC patients, the PBMCs extracted and cultured for 6-days in the presence of phosphopeptide. The cells were then transferred to a prepared IFNγ ELISpot plate and restimulated with phosphopeptide overnight.

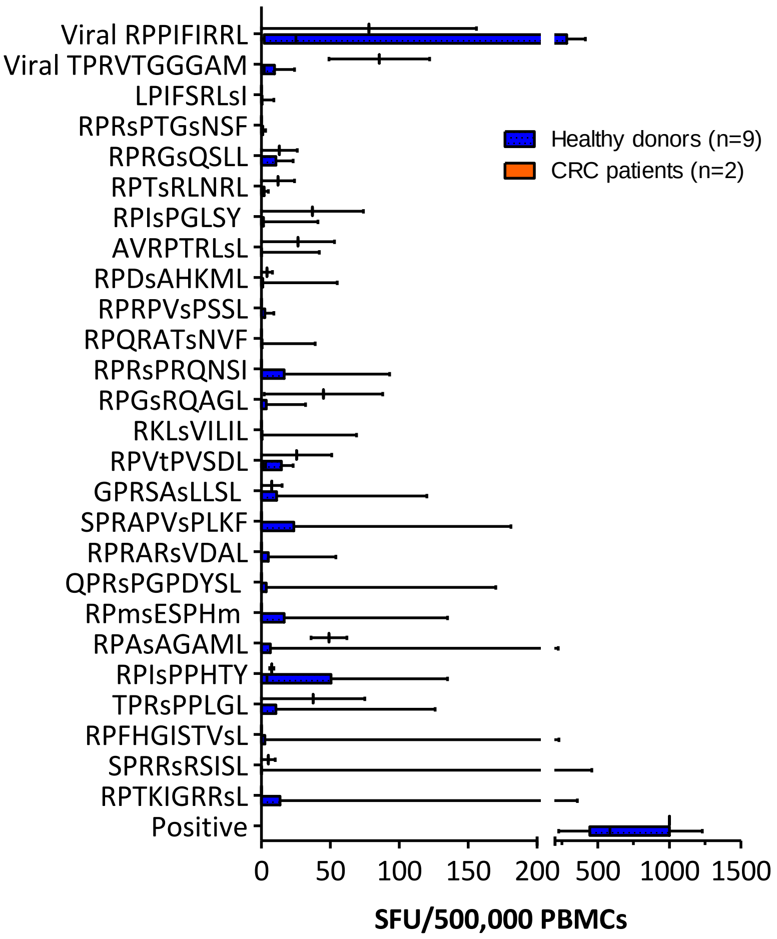

SUPPLEMENTARY FIGURE S11 | T cells targeting the tumor-associated phosphopeptides are found in a number of patients

Comparison of HD and CRC patient PBMC IFNγ production targeting HLA-B*07-associated phosphopeptides. Too few HLA-B*07+ patient samples were obtained for meaningful results, as it is a lower frequency allele, yet there were some responses that were higher in patients than healthy donors.

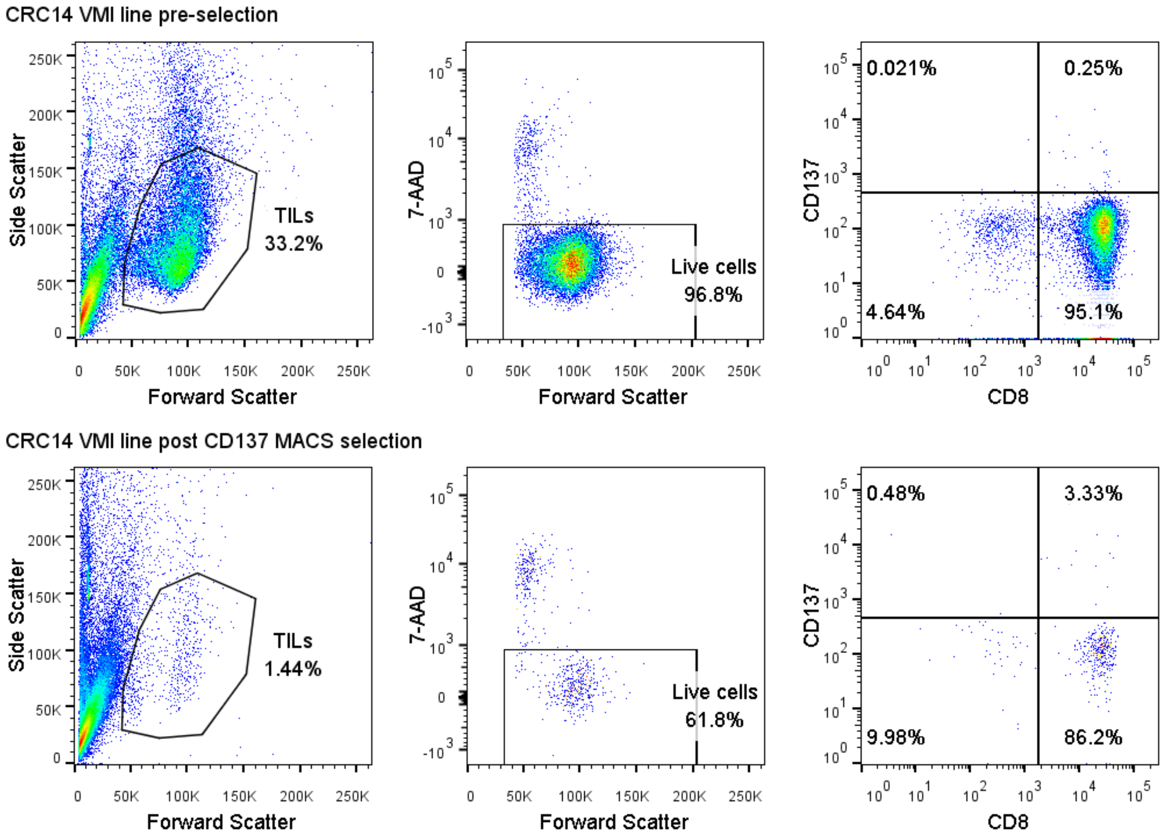

SUPPLEMENTARY FIGURE S12 | CD137 MACS sorting of CRC14

CD8+ TILs were plated at 1E6/ml in TIL medium and stimulated with 10 µg/ml of the VMI (TNS3) phosphopeptide. On days 7 and 10 TILs were adjusted to 5E5/ml and half of the medium exchanged. On day 14 phosphopeptide-specific TILs were selected, using CD137 MACS (Miltenyi biotec). Proir to selection, half of the cells were peptide pulsed for 2 hours, washed and added to the other half overnight. These were then rapidly expanded, as described previously.

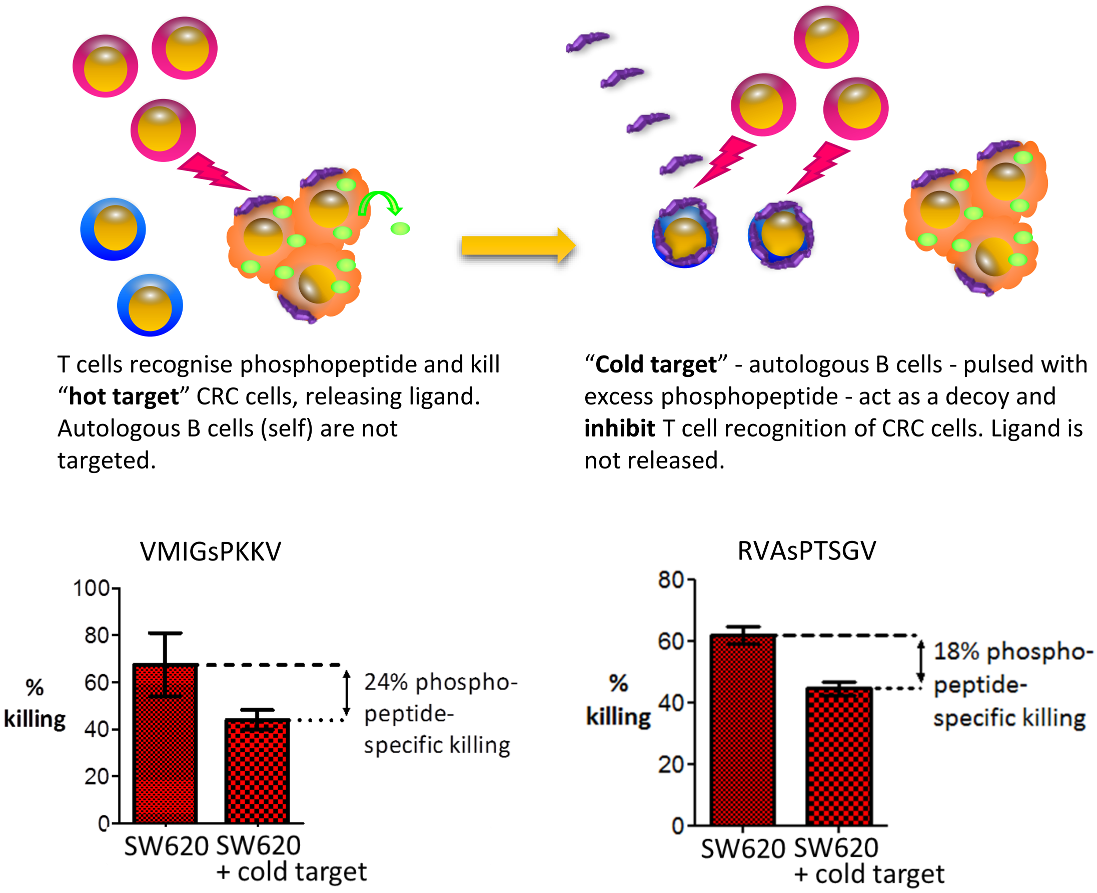

SUPPLEMENTARY FIGURE S13 | Cold-target inhibition to quantify phosphopeptide-specific killing of CRC cell lines

Healthy donor PBMCs were used to establish T cell lines targeting two phosphopeptides – VMIGsPKKV and RVAsPTSGV. After selection and rapid expansion, the lines were used in Europium release killing assays to assess killing of Sw620 cells, which natively express those phosphopeptides. Cold-target inhibition was used to quantify the killing that was targeting the phosphopeptides, as depicted.

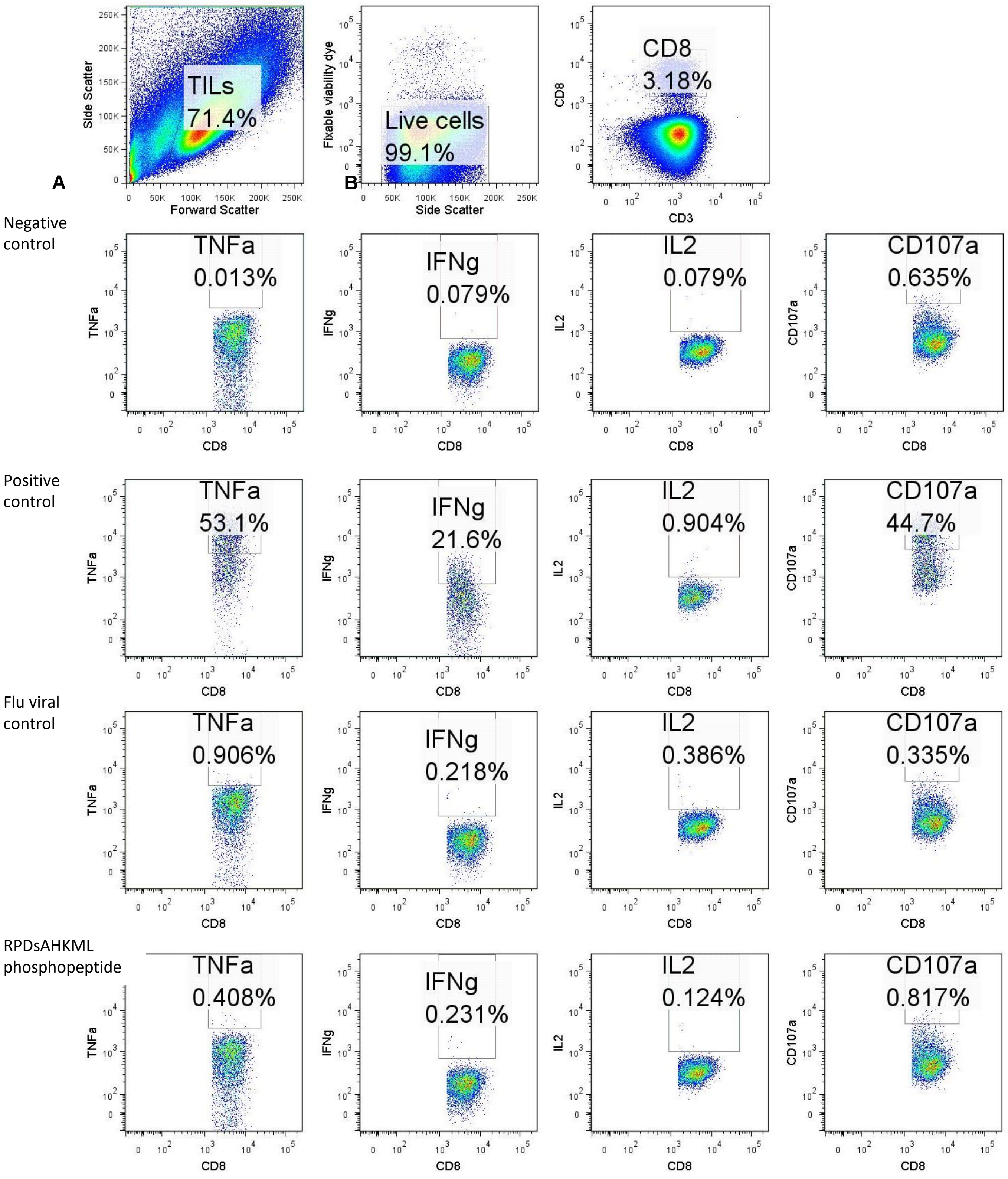

SUPPLEMENTARY FIGURE S14 | Degranulation marker CD107a is upregulated in response to phosphopeptide RPDsAHKML in young CRCLM1 TILs, but not bystander T cells targeting a peptide from influenza.
